# Disruption of Transcriptional Coactivator Sub1 Leads to Genome-Wide Re-distribution of Clustered Mutations Induced by APOBEC in Active Yeast Genes

**DOI:** 10.1371/journal.pgen.1005217

**Published:** 2015-05-05

**Authors:** Artem G. Lada, Sergei F. Kliver, Alok Dhar, Dmitrii E. Polev, Alexey E. Masharsky, Igor B. Rogozin, Youri I. Pavlov

**Affiliations:** 1 Eppley Institute for Research in Cancer and Allied Diseases, University of Nebraska Medical Center, Omaha, Nebraska, United States of America; 2 Department of Genetics and Biotechnology, Saint Petersburg State University, St. Petersburg, Russia; 3 Department of Genetics, Cell Biology and Anatomy, and Munroe-Meyer Institute, University of Nebraska Medical Center, Omaha, Nebraska, United States of America; 4 Research Resource Center for Molecular and Cell Technologies, Research Park, St. Petersburg State University, St. Petersburg, Russia; 5 National Center for Biotechnology Information, National Library of Medicine, National Institutes of Health, Bethesda, Maryland, United States of America; 6 Institute of Cytology and Genetics, Novosibirsk, Russia; 7 Novosibirsk State University, Novosibirsk, Russia

## Abstract

Mutations in genomes of species are frequently distributed non-randomly, resulting in mutation clusters, including recently discovered *kataegis* in tumors. DNA editing deaminases play the prominent role in the etiology of these mutations. To gain insight into the enigmatic mechanisms of localized hypermutagenesis that lead to cluster formation, we analyzed the mutational single nucleotide variations (SNV) data obtained by whole-genome sequencing of drug-resistant mutants induced in yeast diploids by AID/APOBEC deaminase and base analog 6-HAP. Deaminase from sea lamprey, PmCDA1, induced robust clusters, while 6-HAP induced a few weak ones. We found that PmCDA1, AID, and APOBEC1 deaminases preferentially mutate the beginning of the actively transcribed genes. Inactivation of transcription initiation factor Sub1 strongly reduced deaminase-induced *can1* mutation frequency, but, surprisingly, did not decrease the total SNV load in genomes. However, the SNVs in the genomes of the *sub1* clones were re-distributed, and the effect of mutation clustering in the regions of transcription initiation was even more pronounced. At the same time, the mutation density in the protein-coding regions was reduced, resulting in the decrease of phenotypically detected mutants. We propose that the induction of clustered mutations by deaminases involves: a) the exposure of ssDNA strands during transcription and loss of protection of ssDNA due to the depletion of ssDNA-binding proteins, such as Sub1, and b) attainment of conditions favorable for APOBEC action in subpopulation of cells, leading to enzymatic deamination within the currently expressed genes. This model is applicable to both the initial and the later stages of oncogenic transformation and explains variations in the distribution of mutations and *kataegis* events in different tumor cells.

## Author Summary

Genomes of tumors are heavily enriched with mutations. Some of these mutations are distributed non-randomly, forming mutational clusters. Editing cytosine deaminases from APOBEC superfamily are responsible for the formation of many of these clusters. We have expressed APOBEC enzyme in diploid yeast cells and found that most of the mutations occur in the beginning of the active genes, where transcription starts. Clusters of mutations overlapped with promoters/transcription start sites. This is likely due to the weaker protection of ssDNA, an ultimate APOBEC deaminase enzyme target, in the beginning of the genes. This hypothesis was reinforced by the finding that inactivation of Sub1 transcription initiation factor, which is found predominantly in the regions of transcription initiation, leads to further increase in mutagenesis in the beginning of the genes. Interestingly, the total number of mutations in the genomes of Sub1-deficient clones did not change, despite the 100-fold decrease in frequency of mutants in a reporter gene. Thus, the drastic change in genome-wide distribution of mutations can be caused by inactivation of a single gene. We propose that the loss of ssDNA protection factors causes formation of mutation clusters in human cancer.

## Introduction

Faithful replication of genomes and accurate repair of damaged DNA ensures the low mutation rates necessary for the functionality of living cells and organisms. An elevated mutation rate leads to cancer. On the other hand, mutations provide the raw material for evolution on the population level. The tight balance between genome stability and mutagenesis is fundamental to the survival of a species. Errors of replicative polymerases *per se*, as well as failures of replication-coupled mismatch repair (MMR), contribute to mutagenesis on undamaged templates (reviewed in [1, 2]). Additionally, the damage of DNA by both exogenous and endogenous agents leads to miscoding lesions or stalls the replication that promotes the recruitment of less accurate translesion synthesis (TLS) DNA polymerases [3].

One of the major endogenous sources of DNA damage is deamination of cytosine. This reaction can occur spontaneously, but in vertebrates, there are potent catalysts—specialized proteins belonging to the AID/APOBEC superfamily of deaminases playing various, sometimes enigmatic, roles in differentiation, humoral and innate immunity, and a plethora of other processes [4–7]. This group of proteins recently gained fervent attention from cancer biologists, because of the finding of the clusters of mutations (*kataegis*) characterized by APOBEC-like mutation signatures in various human cancers [8–16]. The ability of deaminases to produce clustered mutations, first described *in vitro* [17], is retained in the foreign environment *in vivo* and deaminases robustly induce *kataegis* in model organisms [18–20].

APOBEC proteins catalyze deamination of cytosine to uracil in single-stranded DNA (ssDNA) [17]. The ssDNA-binding proteins, e.g. RPA, attenuate this process [21–23]. Expression of deaminases in the classic work-horses of mutagenesis studies, *E*.*coli* and yeast, elevated mutation frequency in reporter genes [24–27] and caused a genome-wide accumulation of mutations [18, 19, 28]. The major sources of ssDNA in the cells are replication, repair, recombination and transcription, and it is unknown to what extent the ssDNA formed in these processes is accessible for deaminases.

In the current study we induced the expression of APOBEC deaminases in diploid yeast cell cultures approaching saturation and, thus, the cessation of growth. We found that most of the deaminations leading to characteristic cluster-prone mutagenesis occurred in a transcription-dependent manner. Most mutations were present at the beginning of the genes, and inactivation of DNA-binding protein Sub1 involved in the regulation of transcription exaggerated this effect, leading to a striking genome-wide redistribution of mutation densities. Taken together, transcription-dependent cytosine deamination by APOBEC proteins under the conditions of depletion of ssDNA-protecting proteins (such as Sub1) contributes to the induction of multiple clustered mutations in drug-resistant clones in diploid cells. This model can explain the diverse mutation patterns formed during the initiation and progression of cancer.

## Results

### Genomes of PmCDA1-, AID-, APOBEC1- and APOBEC3G-induced drug-resistant mutants in diploids are enriched with SNVs

Previously, we have used sea lamprey deaminase PmCDA1 (*P* *etromyzon* *m* *arinus* cytosine deaminase, [29]) to study the effects of APOBEC proteins on mutagenesis in yeast. Using whole-genome sequencing of deaminase-induced Can^R^ or FOA^R^ (resistant to canavanine and 5-fluoroorotic acid, respectively) clones, we have found a striking genome-wide accumulation of mutations and explained this by a hypothesis of a hypermutable fraction of a diploid yeast cells population where APOBEC is extraordinary active [28]. Strong clustering of deaminase-induced mutations detected in these experiments [18] resembled recently discovered *kataegis*—mutational showers in human cancers [18]. Here, we analyze the mechanisms of the clustering utilizing our yeast system. In addition to the genomes of mutant clones from our previous study [28], we sequenced genomes of a new set of mutant clones (Table 1). These mutants were induced by PmCDA1 expression in diploid LAN210 deficient in Ung1 protein (disruption of *UNG1* gene; Ung, uracil-DNA-glycosylase). The *UNG1* gene encodes for the base excision repair enzyme uracil-DNA-glycosylase, which excises uracil from DNA, thus allowing for unbiased detection of deamination sites in *ung1* strains [24].

**Table 1:** Wild-type strains and mutant clones of yeast used in this work.

Mutagen	Parental strain	Day	Selection	Clone systematic name	Source	Number of SNVs
None	LAN200 ref	-	-	N001-LAN200-wt-NA-NA-RUN1	[28]	-
None	LAN201 ref	-	-	N002-LAN201-wt-NA-NA-RUN1	[28]	-
None	LAN210 ref	-	-	N008-LAN210-wt-NA-NA-RUN2	[28]	-
PmCDA1	LAN210	3	Can^R^	N010-LAN210-Can-PmCDA1-NA-RUN2-D3	[28]	1236
PmCDA1	LAN210	3	Can^R^	N011-LAN210-Can-PmCDA1-NA-RUN2-D3	[28]	385
PmCDA1	LAN210	3	Can^R^	N012-LAN210-Can-PmCDA1-NA-RUN2-D3	[28]	940
PmCDA1	LAN210	3	Can^R^	N013-LAN210-Can-PmCDA1-NA-RUN2-D3	[28]	1863
PmCDA1	LAN210	3	Can^R^	N058-LAN210-Can-PmCDA1-Feb13-RUN5-D3	[28]	453
PmCDA1	LAN210	3	Can^R^	N061-LAN210-Can-PmCDA1-NA-RUN5-D3	[28]	550
PmCDA1	LAN210	3	Can^R^	N062-LAN210-Can-PmCDA1-NA-RUN5-D3	[28]	514
PmCDA1	LAN210	3	FOA^R^	N059-LAN210-FOA-PmCDA1-Feb13-RUN5-D3	[28]	815
PmCDA1	LAN210	3	FOA^R^	N060-LAN210-FOA-PmCDA1-Feb13-RUN5-D3	[28]	989
PmCDA1	LAN210	3	Can^R^	N065-LAN210-Can-PmCDA1-NA-RUN6-D3	This work	705
PmCDA1	LAN210	3	Can^R^	N066-LAN210-Can-PmCDA1-NA-RUN6-D3	This work	499
PmCDA1	LAN210 *sub1*::*KanMX*	3	Can^R^	N067-LAN210_sub1KanMX-Can-PmCDA1-NA-RUN6-D3	This work	1966
PmCDA1	LAN210 *sub1*::*KanMX*	3	Can^R^	N068-LAN210_sub1KanMX-Can-PmCDA1-NA-RUN6-D3	This work	1185
PmCDA1	LAN210 *sub1*::*KanMX*	3	Can^R^	N069-LAN210_sub1KanMX-Can-PmCDA1-NA-RUN6-D3	This work	5361
APOBEC1	LAN210	3	Can^R^	N034-LAN210-Can-A1-Oct12-RUN4-D3	This work	208
APOBEC1	LAN210	3	Can^R^	N035-LAN210-Can-A1-Oct12-RUN4-D3	This work	160
APOBEC1	LAN210	3	FOA^R^	N036-LAN210-FOA-A1-Oct12-RUN4-D3	This work	216
APOBEC1	LAN210	3	FOA^R^	N037-LAN210-FOA-A1-Oct12-RUN4-D3	This work	237
APOBEC3G	LAN210	3	Can^R^	N038-LAN210-Can-A3G-Oct12-RUN4-D3	This work	73
APOBEC3G	LAN210	3	Can^R^	N039-LAN210-Can-A3G-Oct12-RUN4-D3	This work	65
AID	LAN210	3	Can^R^	N040-LAN210-Can-AID-Oct12-RUN4-D3	This work	589
AID	LAN210	3	Can^R^	N041-LAN210-Can-AID-Oct12-RUN4-D3	This work	114
HAP	LAN211	N/A	Can^R^	N006-LAN211-Can-HAP-NA-RUN1	[28]	945
HAP	LAN211	N/A	Can^R^	N007-LAN211-Can-HAP-NA-RUN1	[28]	1554
HAP	LAN211	N/A	Can^R^	N014-LAN211-Can-HAP-NA-RUN2	[28]	1200
HAP	LAN211	N/A	Can^R^	N015-LAN211-Can-HAP-NA-RUN2	[28]	1338
HAP	LAN211	N/A	Can^R^	N016-LAN211-Can-HAP-NA-RUN2	[28]	1164
HAP	LAN211	N/A	Can^R^	N017-LAN211-Can-HAP-NA-RUN2	[28]	1268
HAP	LAN211	N/A	Can^R^	N018-LAN211-Can-HAP-NA-RUN2	[28]	1134
HAP	LAN211	N/A	Can^R^	N019-LAN211-Can-HAP-NA-RUN2	[28]	1120
HAP	LAN211	N/A	Can^R^	N050-LAN211-Can-HAP-NA-RUN4	[28]	1142
HAP	LAN211	N/A	Can^R^	N051-LAN211-Can-HAP-NA-RUN4	[28]	1812
HAP	LAN210 *sub1*::*KanMX*	N/A	Can^R^	N081-LAN210_sub1KanMX-Can-HAP-NA-RUN6	This work	1172
HAP	LAN210 *sub1*::*KanMX*	N/A	Can^R^	N082-LAN210_sub1KanMX-Can-HAP-NA-RUN6	This work	3504

Genomes of PmCDA1-induced mutants were highly enriched with mostly heterozygous GC->AT transitions SNVs, as expected from cytosine deamination in the *ung1* background (see Table 1 for the list of sequenced mutants and S1 Dataset for SNV datasets; also see [28]). We have also sequenced the genomes of limited numbers of mutants induced by human deaminase AID, rat APOBEC1, and human APOBEC3G. These deaminases induced less mutations in the genome, consistent with the established PmCDA1 superior deaminase activity among the other APOBEC members [27]. Still, genomes of these AID-, APOBEC1- and APOBEC3G-induced mutants possess hundreds of SNVs (Table 1).

**Fig 1:**
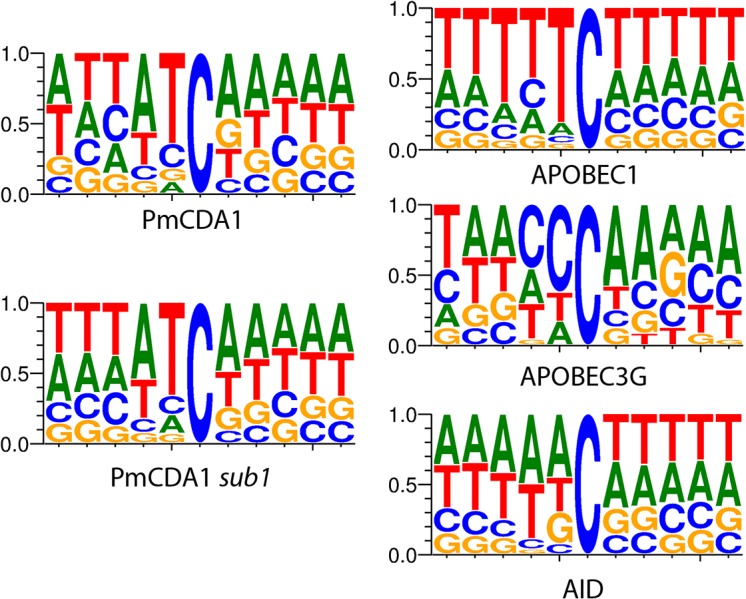
APOBEC deaminases induce mutations in yeast in the characteristic sequence contexts. Sequence logos created from pooled data and normalized for yeast GC content (38%) are shown. Y-axis, probability of corresponding nucleotide.

We have also analyzed the sequence context of base substitutions induced by APOBECs in the yeast genome (Fig 1). Clearly, the classical sequence preferences of deaminases are maintained in yeast cells (see Discussion).

### Multiple clusters of mutations are detected in genomes of mutants induced by PmCDA1

**Fig 2:**
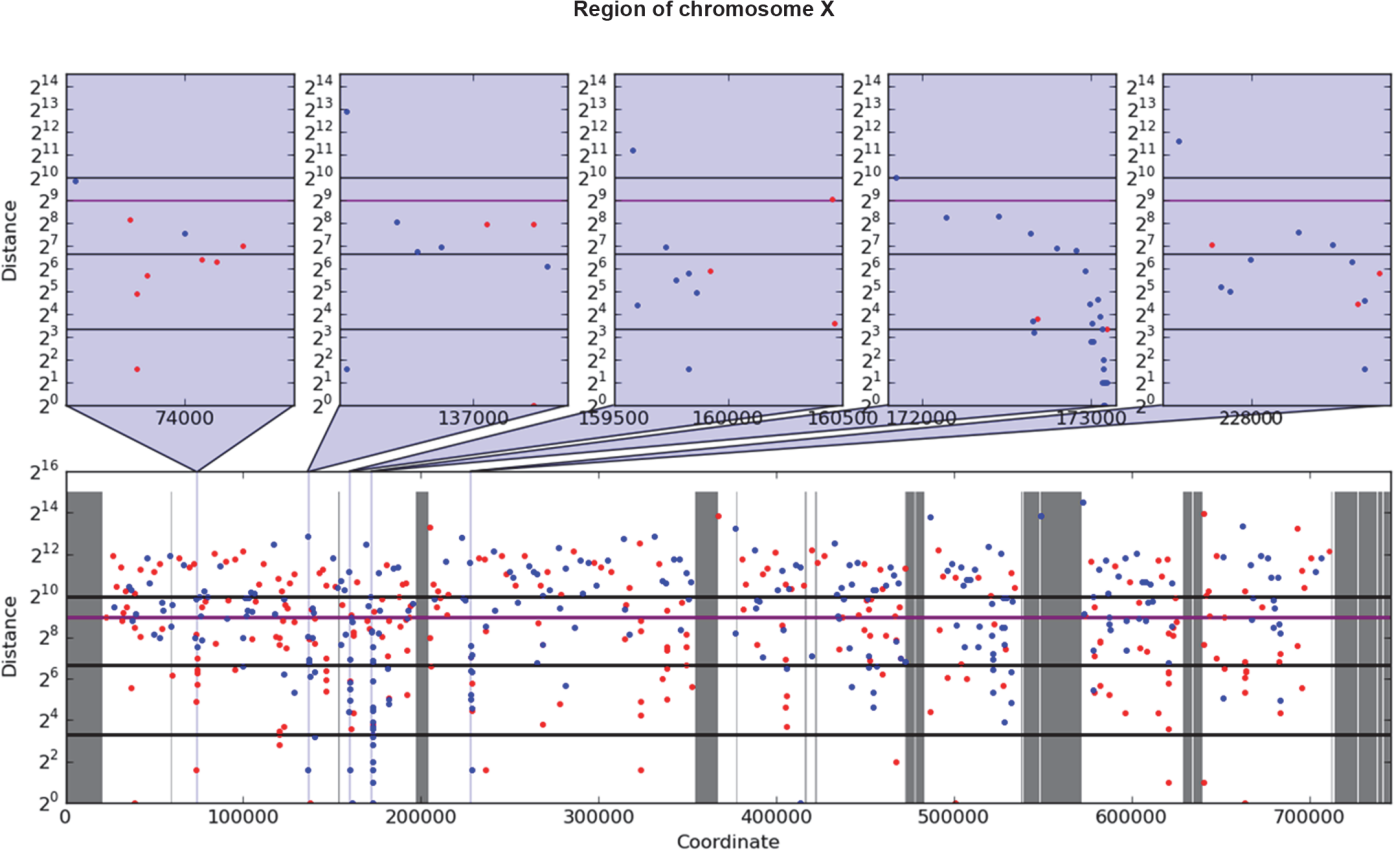
Example of rainfall plot showing mutation clusters in PmCDA1-induced mutants. Whole chromosome X is shown at the bottom. The most interesting clusters are shown at the top. X-axes, chromosomal coordinate in bp; Y-axes, distance from the mutation to the previous SNV, bp (log_2_ scale). Each dot represents a single SNV. Red, C->T, blue, G->A. Vertical lines: dark grey, gaps in the reference genome; light grey, masked regions; blue, position of clusters shown on the top panel. Thick horizontal lines (from bottom to top) mark 10, 100, 500 (magenta), and 1000 values.

For analysis of mutation clustering, we have used the pool of SNVs induced by PmCDA1 in the LAN210 strain. For a comparison, we used the pool of SNVs from the genomes of Can^R^ mutants induced by 6-hydroxylaminopurine (6-HAP) ([28], Table 1), a chemical mutagen that produces a much more random distribution of mutations [18, 30, 31]. First, we have applied a convenient visualization tool, a rainfall plot. We have slightly modified the original rainfall plot design [8]: instead of the mutation number, the X-axis contains chromosomal coordinates. We have also used a log_2_ scale on the Y-axis. PmCDA1-induced clusters were observed throughout the yeast genome (S1 Fig). Much less clustering was observed with the HAP data. An example of mutation clustering on chromosome X in PmCDA1-induced mutants is shown on Fig 2.

Next, we performed a more rigorous and unbiased identification of clusters. Based on hierarchical cluster analysis, we developed a new method of detection of mutation clusters (Materials and Methods). Using this approach, we detected multiple clusters of mutations in the genomes of mutants induced by both PmCDA1 and HAP. The set of SNVs can be divided into several subsets (clusters), if the distances between SNVs inside the cluster are less than the distance between it and neighbor clusters. Depending on the mutational densities, different cluster sizes can be obtained during such a classification. For example, two closely located groups of mutations can be classified as two small clusters or one big cluster. Using different values of a threshold for extracting clusters from hierarchical clustering, we have found that the increase in the number of clusters with three or more mutations reaches or nearly reaches a plateau when the threshold was set to a value in the range of 1,000 to 2,000 bp (S2 Fig). Considering the high density of mutations in our dataset, and *a priori* knowledge of the approximate cluster size (see rainfall plots on Fig 2), we have set the cutoff of the cophenetic distance for extraction to 1,000 bp (see Materials and Methods for more details on clustering analysis).

**Fig 3:**
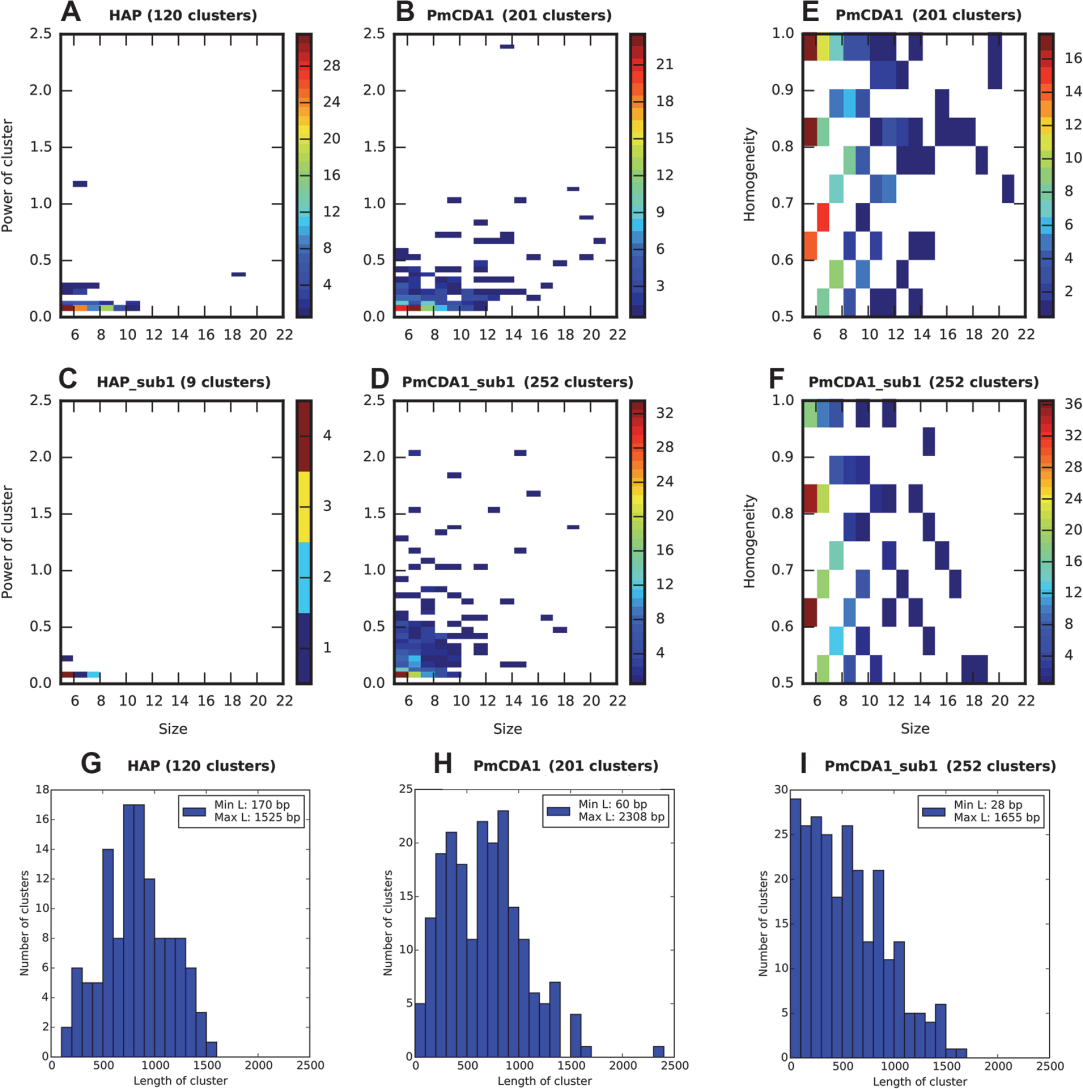
PmCDA1 induces diverse clusters of mutations in diploid yeast genome. Clusters with size> = 5, and power> = 0.05, are shown. For all panels, the strains (*SUB1* or *sub1*) and mutagen are indicated. A-D. Heatmap diagrams representing clusters of different size (X axis) and power (Y-axis). E-F. Heatmap diagrams showing dependence of PmCDA1 cluster homogeneity (Y axis) and size (X axis). For heatmaps (panels A-F), the color of the blocks represent the number of clusters with corresponding parameters. Color scale is shown on the right side of every panel. G-I. Distribution of cluster lengths (bp) for HAP and PmCDA1 in *SUB1* and *sub1* strains. Bin size, 100 bp.

Each cluster of mutations is characterized by its size (number of SNVs) and power (number of SNVs/median of distances between mutations within the cluster). The distributions of clusters of different size and power are shown in Fig 3 (on this and the following figures we present the data for both *SUB1+* and *sub1* mutant strains; see the last section of Results for the information and rationale for using *sub1*). By defining cutoff values for the cluster size and power, we can detect a different number of clusters by including the most powerful clusters in the analysis and discarding the “background” ones (these can be due to random SNV neighboring, considering high mutational density in our dataset). On the Fig 3A–3D, numbers of clusters (size> = 5, power> = 0.05) of different size and power are shown for the different strains, for both PmCDA1 and HAP. Clearly, PmCDA1 induced more clusters than HAP, and PmCDA1 clusters were more powerful (Fig 3A and 3B). The distributions of cluster size are also shown (Fig 3G–3I).

Analysis of mutation types within the clusters revealed different types of clusters (Fig 3E and 3F). Polar clusters consist exclusively of C->T or G->A mutations and represent deamination events occurring by an action of deaminase on the DNA strand of the same orientation (either Watson or Crick in relation to the reference genome sequence; see [32] for convention explaining DNA strands names). Since our cells are diploid, deamination can happen in different chromosomes; however, the strand polarity holds in the case of polar clusters. Mixed clusters possessed both C->T and G->A mutations and therefore represent deaminations in different strands of the DNA duplex spanning the region of the cluster. We defined homogeneity as a measure of cluster polarity. A cluster with homogeneity = 1 consists of SNVs of the same type (either C->T or G->A); homogeneity = 0.5 means that the cluster possesses 50% C->T and 50% G->A mutations. Clusters with homogeneity between 0.5 and 1.0 represent deviations from perfectly polar clusters (1.0) and non-polar ones (0.5) (see Materials and Methods).

**Fig 4:**
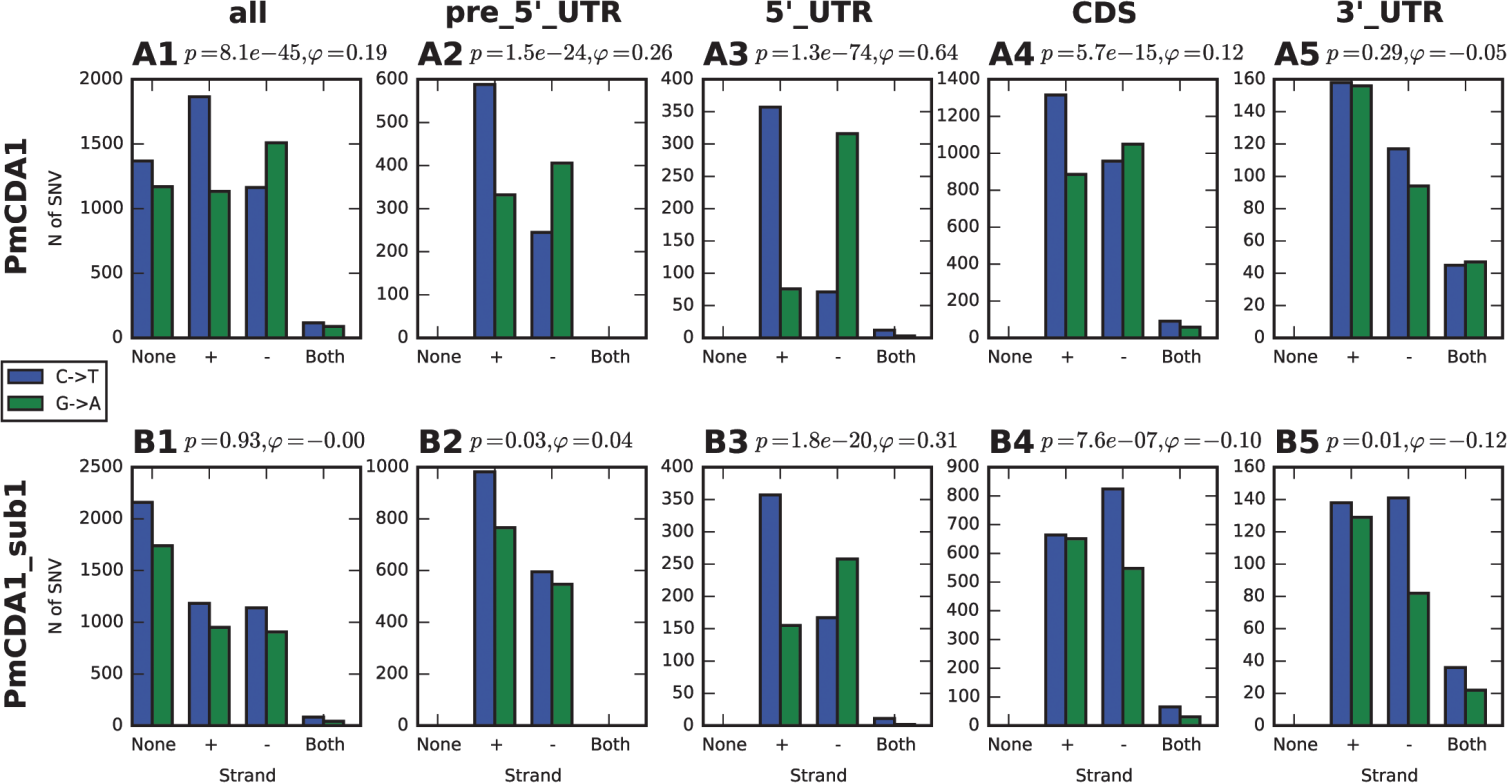
Analysis of strand-specificity of mutations reveals the predominant deamination of non-transcribed DNA strand and attenuation of this effect in strains defective in Sub1. Strain genotype and type of genomic feature are indicated. Blue, C->T, Green, G->A. None, mutation does not hit any genomic feature; (+), mutation is found in the gene in the Crick strand; (-), mutation is found in the gene in the Watson strand; and both, mutation is found in the region where genes in Watson and Crick strand overlap. Phi, correlation coefficient; phi>0 indicates strand-specificity so that C->T mutations preferentially hit the non-transcribed strand; phi<0 indicates opposite strand specificity; *p*-values are shown.

Clusters with the homogeneity values close to 1, including completely polar clusters, can arise as the intermediates of transcription and replication, as well as recombination, when only one of the DNA strands is exposed and available for APOBEC. Since our mutants are selected from the three-day saturated culture (see Materials and Methods), we hypothesized that deamination is occurring during transcription. The non-transcribed strand in the transcriptional bubble is exposed and particularly vulnerable to the deaminase ([33], and see Discussion). Indeed, we found an enrichment in the C->T mutations in the genes located in the Watson strand of the genome; complementary, more G->A mutations have been found in the genes oriented in the Crick strand (Fig 4A1).

### Most clusters are found in the beginning of transcriptionally active genes

**Fig 5:**
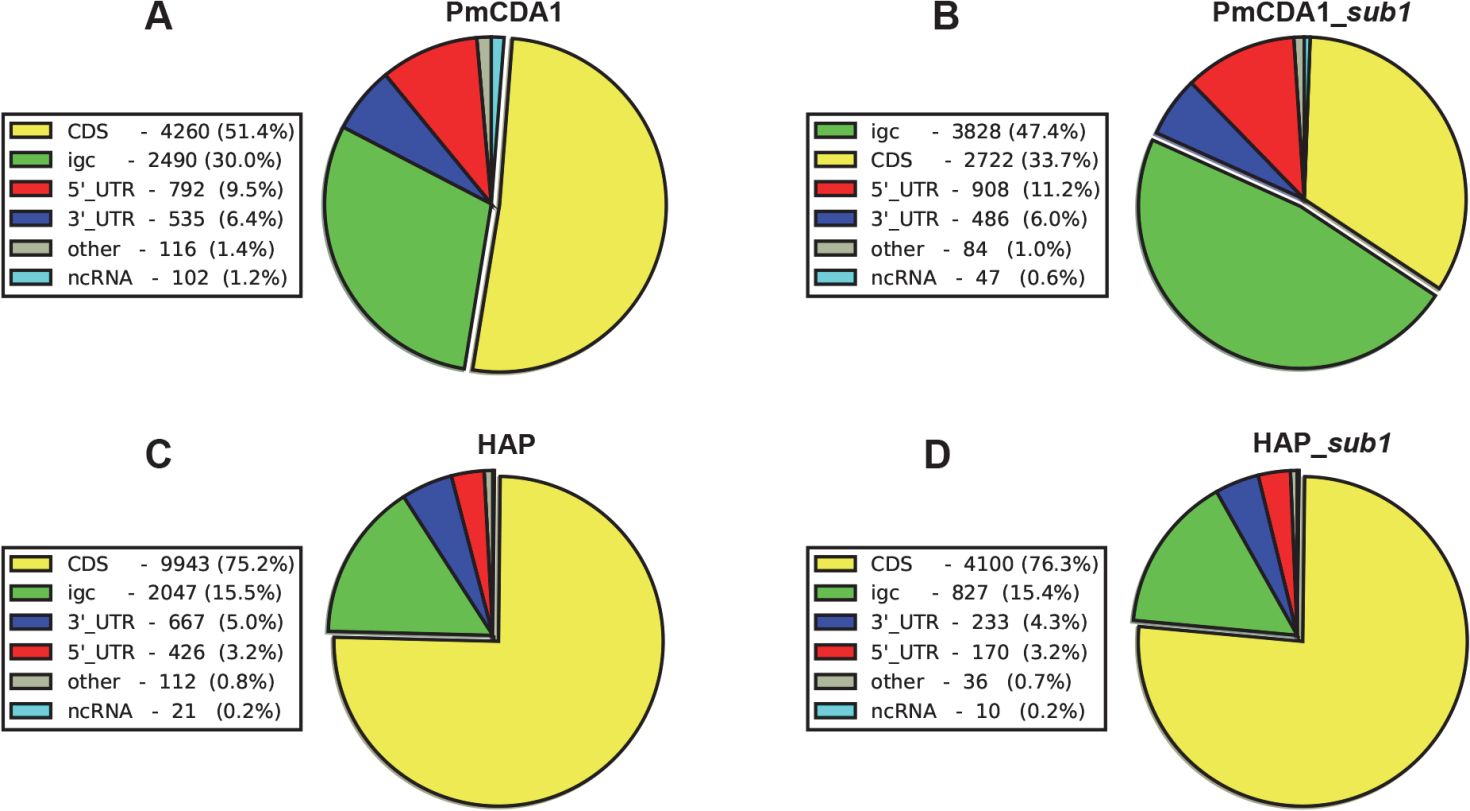
Deaminases induce more mutations in intergenic and 5’-UTRs than in protein-coding regions. Proportions of SNVs found in different genomic features are represented as a pie chart. igc, intergenic. A. PmCDA1. B. PmCDA1, *sub1* strain. C. 6-HAP. D. 6-HAP, *sub1* strain.

Previously, we have found that PmCDA1 is inducing more than the expected mutations outside the open reading frames (ORF) [28]. We have confirmed and elaborated these findings (Fig 5) by annotating our reference genome using RNA sequencing data obtained in [34], which allowed us to map 5’- and 3’-UTRs (untranslated regions) for most of the genes (see Materials and Methods). To our knowledge, LAN210 is the first yeast reference genome with annotated UTRs in the “gold standard” GFF annotation format. PmCDA1 induced more mutations in the regions annotated as intergenic, 5’-UTR, and ncRNA (non-coding RNA), as compared to 6-HAP, where the proportion of SNVs roughly reflects the relative abundance of the corresponding features in the yeast genome (Fig 5).

**Fig 6:**
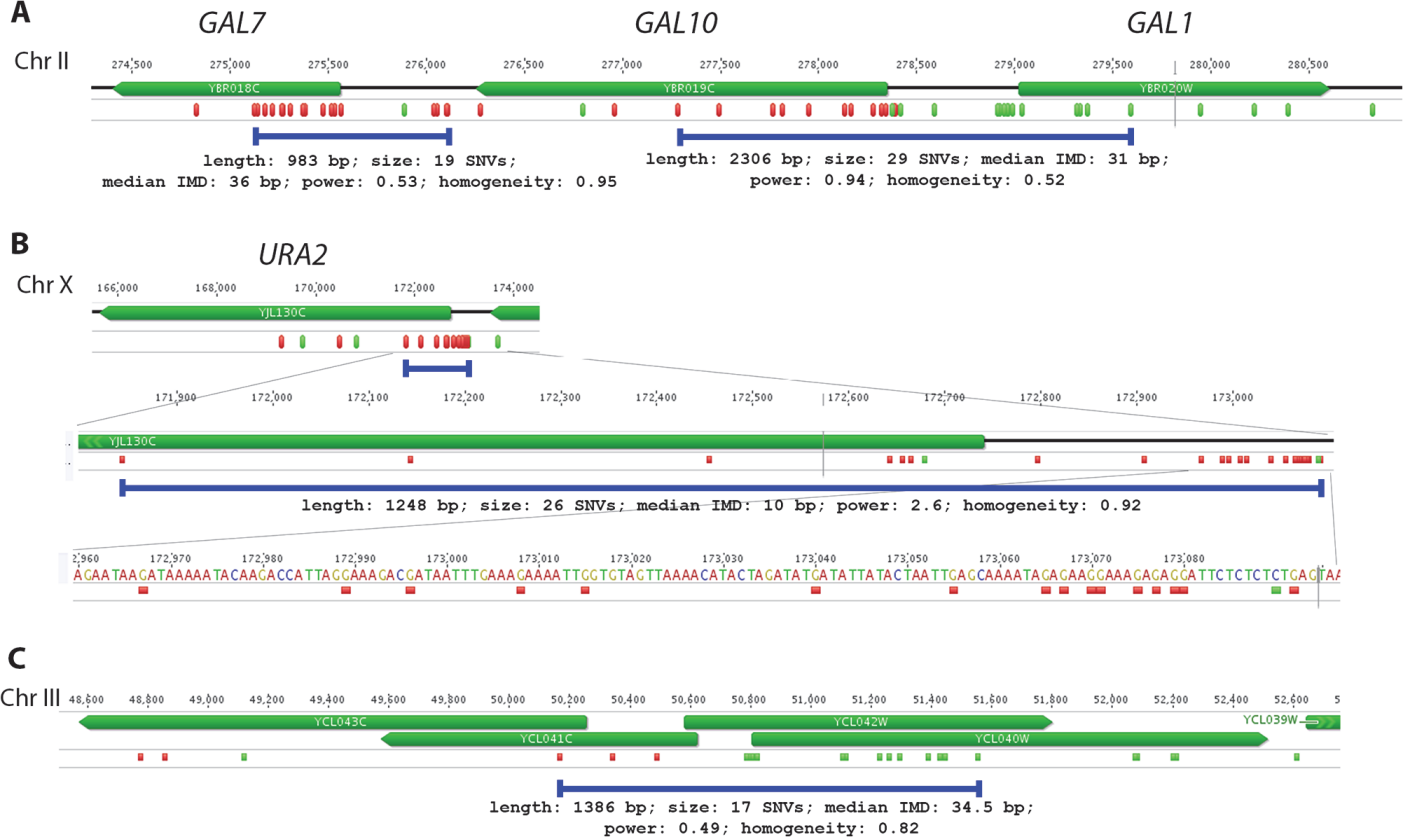
Examples of PmCDA1-induced transcription-associated mutation clusters. A. Cluster of galactose genes. B. The strongest cluster in the genome. C. Cluster within the group of overlapping genes. Green, C->T; Red, G->A.The clusters detected in these genomic regions in the wild-type (*SUB1*) strain are shown as blue lines, and their parameters are indicated. For the *URA2* gene (panel B), the mutations and the cluster are shown in three different scales.

**Fig 7:**
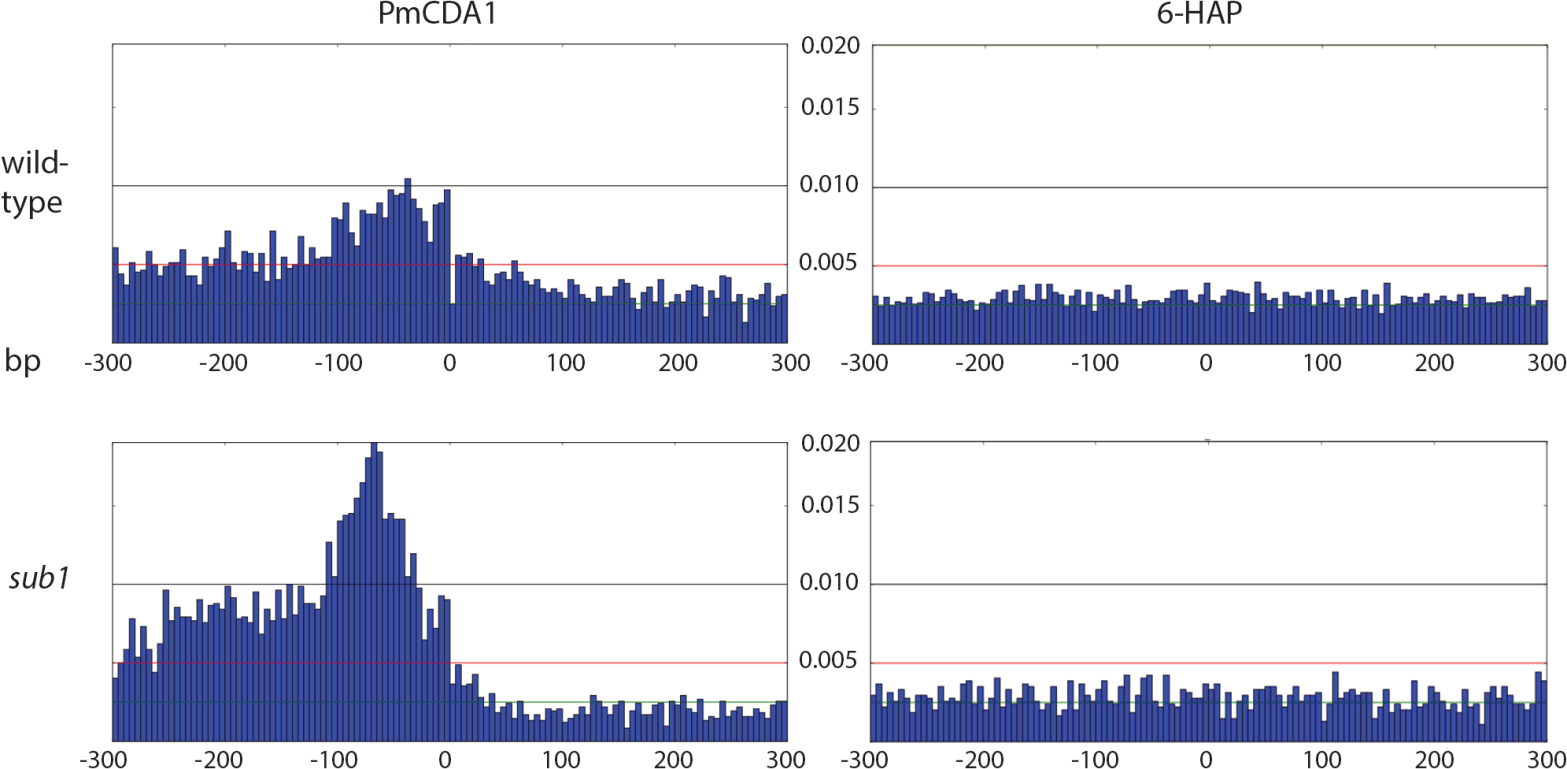
Peak of mutational density before the CDS start in genomes of PmCDA1-induced mutants. Genotypes of strains and mutagen are indicated. X-axis, nucleotide position (bp) from CDS start codon (zero). Y-axis, fraction of mutations through the whole genome belonging to the corresponding 5-bp bins.

We have visually examined the most prominent clusters (Fig 6). Two powerful clusters were detected in the region of chromosome II, where the group of genes responsible for galactose metabolism are located (Fig 6A). This was expected since we grow our cultures in galactose media to induce deaminase expression. The strongest cluster in the genome was found between the *URA2* and *TRK1* genes on the chromosome X (Fig 6B and [18]). Another example is a cluster found on chromosome III, where confirmed and dubious ORFs are present in different orientations (Fig 6C). Some clusters overlap with single gene (such as clusters near the *GAL7* and *URA2* genes, see Fig 6); other clusters span two genes and the corresponding intergenic distance, as in case of clusters near *GAL10* and *GAL1* genes (Fig 6A), and a group of genes shown on Fig 6C. Importantly, the homogeneity of the clusters spanning one gene was higher than for the clusters spanning two genes in the opposite orientation (Fig 6). The visual examination of the pattern of SNVs led to the hypothesis that deaminase preferentially targets the beginning of the genes (Fig 6). To test this, we have plotted mutation densities around the open reading frame (ORF) start codons (see Materials and Methods). We found a peak of mutations around the beginning of the ORF, in the ~100 bp window upstream of the start codon (Fig 7). Further upstream from the ORF start, the SNV density was ~2x lower, and it was ~4x lower in the coding sequence. These effects were also observed for other deaminases—AID, APOBEC1 and APOBEC3G (S3 Fig). No biases were detected in the distribution of HAP-induced mutations (Fig 7). It is important to note that we used all mutations for this analysis, irrespective of whether they belong to the extracted cluster or not.

**Fig 8:**
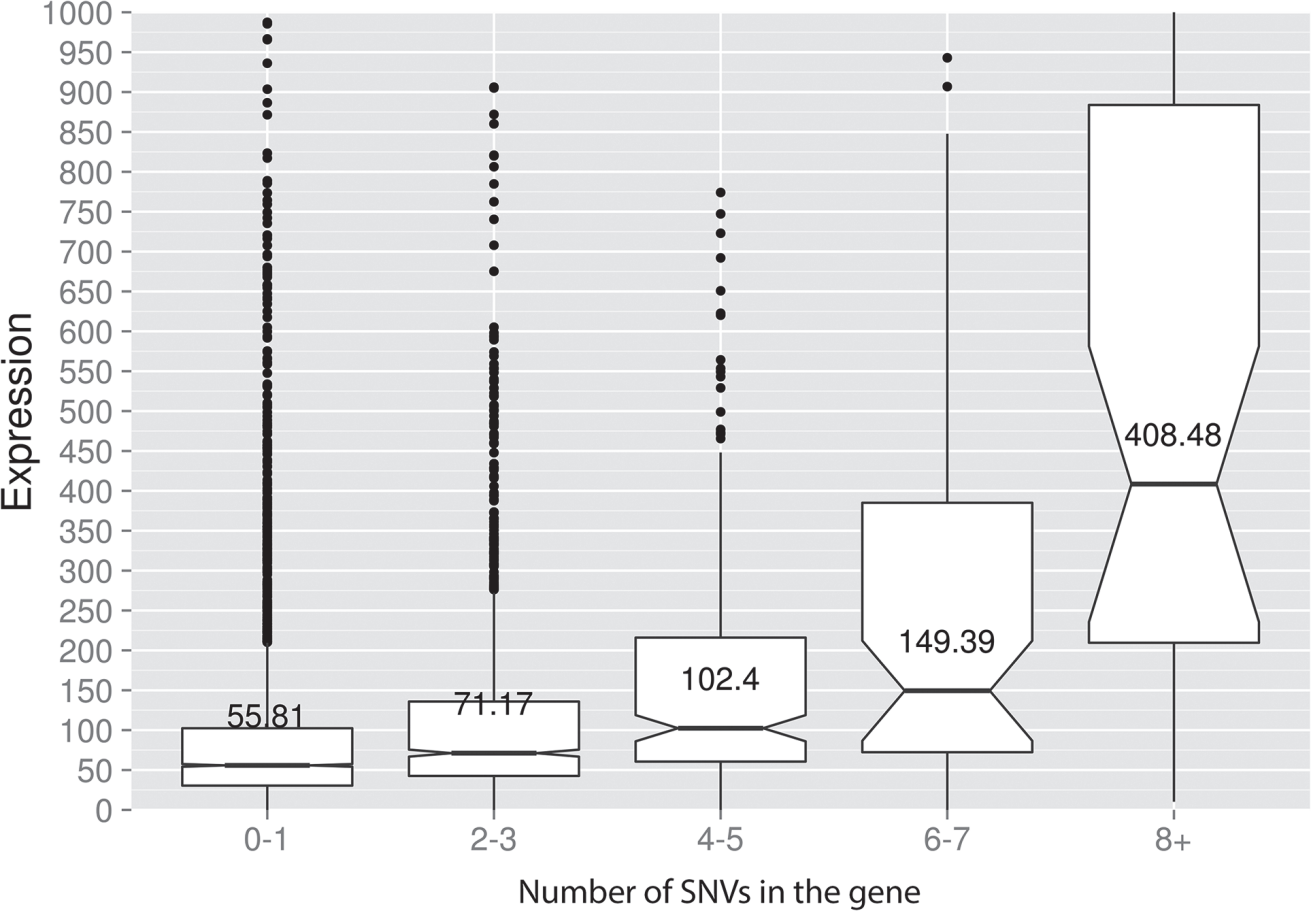
Genes with incrementing mutational load are expressed at increasingly higher levels. Median expression values (FPKM, Y-axis) are shown for the genes possessing different numbers of SNVs (X-axis) in the PmCDA1 dataset. The upper and lower "hinges" correspond to the first and third quartiles (the 25th and 75th percentiles). The upper whisker extends from the hinge to the highest value that is within 1.5 * IQR of the hinge, where IQR is the inter-quartile range. The lower whisker extends from the hinge to the lowest value within 1.5 * IQR of the hinge. Data beyond the end of the whiskers are outliers and plotted as points. The notches extend 1.58 * IQR / sqrt(n). This gives a roughly 95% interval for comparing medians. Some of the outliers are very far from the median values on the linear scale; see S5 Fig for log-transformed plots. “Wild-type” (*SUB1*) strain, two RNA-Seq replicates are averaged.

Taken together, the data presented at this point suggest that deaminases can access DNA and catalyze C->U conversion in the transcriptional bubble. To directly test this hypothesis, we have performed RNA sequencing to determine the transcriptional profile of yeast cells under the conditions of our experiment. We have isolated total RNA from yeast cultures with PmCDA1 being induced, depleted it for rRNA, and sequenced (see Materials and Methods for details of library preparation, sequencing and data analysis). There were no significant differences in RNA expression profiles between biological replicates and between wild-type and *sub1* strains (S4 Fig). However, we have found that genes with mutations are expressed at a higher level (median FPKM for genes with mutations 67.6, without mutation—52.7; Wilcoxon-Mann-Whitney test p = 2.2E-16). Moreover, median expression levels (FPKM) of the genes with increased numbers of SNVs were progressively higher (Figs 8 and S5).

### Transcription initiation factor Sub1 affects the genome-wide profile of enzymatic deamination

What is the cause of preferred deamination in the beginning of the genes? One possibility is the weaker protection of ssDNA in these regions. Sub1 is a transcription factor that binds ssDNA in the regions of transcription initiation. During elongation, Sub1 is substituted for RPA [35], which is known to strongly protect ssDNA from APOBECs [21–23]. We reasoned that Sub1, like RPA, may also protect DNA in the beginning of the genes, albeit less efficiently. To test this hypothesis, we have constructed diploid strain LAN210 *sub1* with disruption of the *SUB1* gene (see Materials and Methods), transformed it with PmCDA1-expressing plasmid and performed a mutagenesis experiment as described in Materials and Methods. Can^R^ mutation frequency in the LAN210 *sub1* strain was 1.62x10^-7^ (95% confidence interval 2.62x10^-8^–3.79x10^-7^), i.e. almost 100 times lower as compared to the LAN210 strain (1.65x10^-5^ (1.46x10^-5^–2.02x10^-5^)). Clearly, disruption of the *SUB1* gene causes a very strong reduction in the number of *can1* mutants.

**Fig 9:**
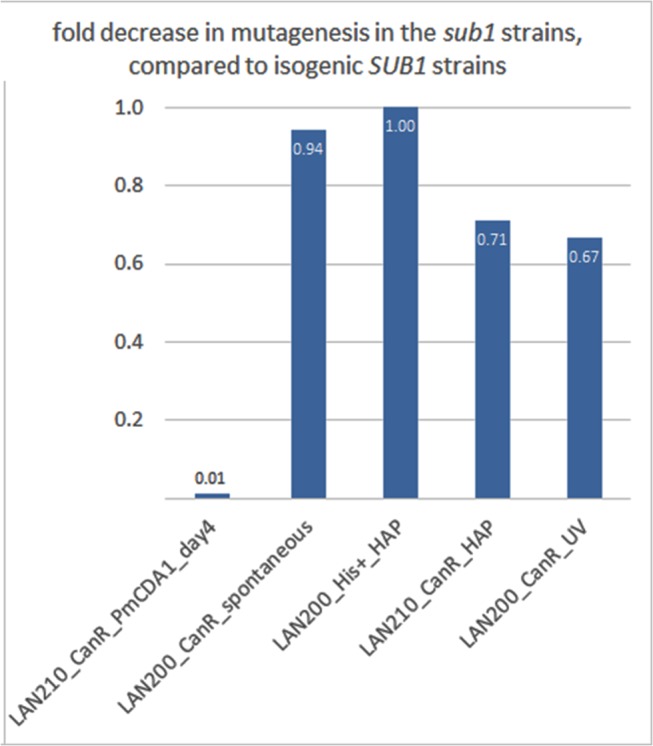
*SUB1* disruption specifically suppresses APOBEC-induced mutagenesis at the *can1* gene. A fold decrease in mutagenesis in diploid (LAN210) or haploid (LAN200) *sub1* strains, compared to the corresponding wild-type. Calculations are based on mutation frequency for PmCDA1, and on the results of a spot-test for spontaneous or 6-HAP or UV-induced mutagenesis.

To check whether the suppressive effect of *sub1* disruption on mutation frequency is specific for deaminases, we have estimated the generation of mutants in the LAN210 *sub1* strain with HAP and UV in a semi-quantitative spot test (see Materials and Methods). No significant differences have been observed in the numbers of spontaneous, HAP-, and UV-induced mutants between wild-type and *sub1* yeast, either haploid or diploid (Figs 9 and S6).

We then sequenced the genomes of *can1* mutant clones induced by PmCDA1 in the LAN210 *sub1* strain (see Table 1). Surprisingly, we did not find any reduction of mutation load per genome in these clones (Table 1). Moreover, one of these clones (N069) possessed 5361 SNVs (~1 mutation per 2 Kb on average), a number that is far higher than in any of the other sequenced clones. Clearly, the reduction in *can1* mutation frequency in the *sub1* strain is not due to the decrease in total number of deaminations in the genome. We also sequenced two genomes of mutants induced in the LAN210 *sub1* by HAP. The numbers of SNVs per genome were similar to the HAP-induced clones with wild-type *SUB1*.

Clustering analysis indicated that the clusters of PmCDA1-induced mutations in *sub1* clones are shorter, smaller in size (number of SNVs in cluster), but more powerful (Fig 3, compare H and I, B and D). Conversely, HAP induced much less clusters in the *sub1* genomes (Fig 3, compare A and C). This effect was true even after normalization for the total number of mutations in all HAP mutants (2.7x more SNPs in the wild-type as compared to *sub1*).

Next, we decided to check whether there is an effect of *SUB1* inactivation on the distribution of mutation densities. In *sub1* strain, the main peak of PmCDA1-induced mutations (100 bp windows upstream of the start codon, see Fig 7) becomes two-fold stronger, and a second peak appears, from about -250 to -100 bp from the start codon. SNV density in the second peak was as high as in the main peak in the wild-type strain. At the same time, there was around two-fold reduction in mutation density in the coding sequences in the *sub1* clones (Fig 7).

**Fig 10:**
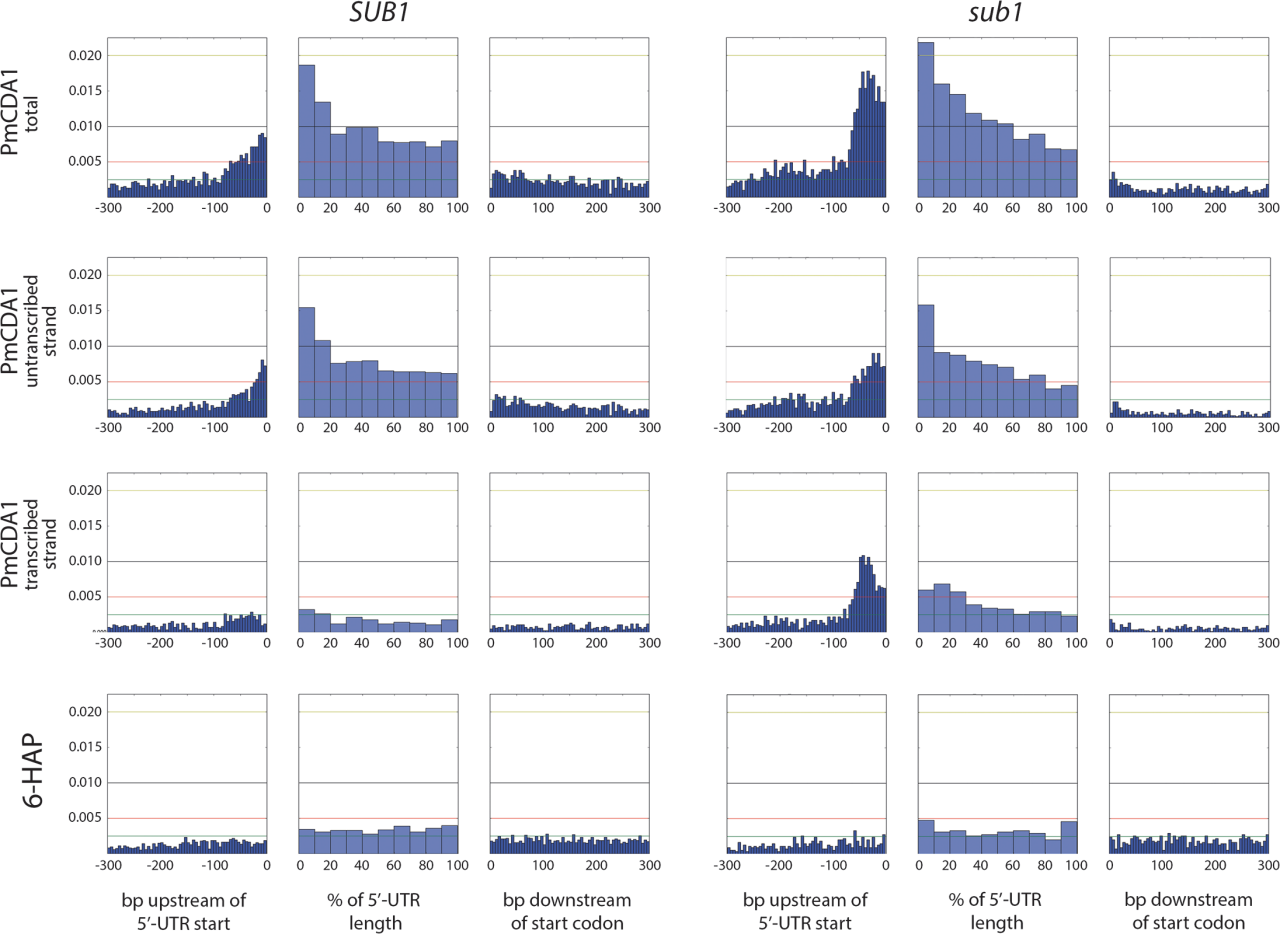
The deletion of *SUB1* leads to a redistribution of mutation densities toward 5’-UTRs and pre-5’-UTR regions in a strand-specific manner. Densities of mutations in the pre-5’-UTR regions, 5’-UTRs, and beginning of coding sequences are presented. Genomic features, as well as the type of mutagen and the genotype of the strain (*SUB1* or *sub1*), are shown. For PmCDA1, densities of mutations resulting from deaminations in the transcribed and non-transcribed strands of genes are shown, as well as cumulative plots (PmCDA1 total). For the 5’-UTRs and pre-5’-UTRs, only SNVs that do not hit the overlapping genomic feature are shown. The 5’-UTRs shorter than 10 bp are excluded from the analysis. X-axis: for pre-5’-UTR, bp from the 5’-end of 5’-UTR (bin size 5 bp); for 5’-UTR, percent of 5’-UTR length (bin size 10%); for CDS, bp from the start codon (bin size 5 bp); Y-axis: the fraction of mutations through the whole genome belonging to the corresponding bins.

We have also analyzed the correlation between the transcription start site (TSS) and the mutation density peak. In Fig 10, we have plotted mutation densities 300 bp upstream of the 5’-UTR start, 300 bp downstream of the start codon, and in the 5’-UTR with normalization on the UTR length. The design of these plots allows for simultaneous visualization of the transcription start site and the ORF start, which would be otherwise impossible due to high variability in the 5’-UTR lengths. In the wild-type strain, the mutation density peak exactly corresponds to the position of the transcription start site, with mutations being introduced both into the transcribed region (downstream of TSS), and the untranscribed DNA (upstream of TSS) (see Fig 10, first row of plots, left side). In the *sub1* strain, the peak slightly shifts upstream from the TSS (Fig 10, first row of plots, right side). No significant deviations from the random mutation distribution was observed in case of 6-HAP (Fig 10, bottom row of plots).

We also analyzed the distribution of mutations relative to the strand (transcribed on non-transcribed) that undergoes deamination (Fig 10, second and third rows of plots). This analysis revealed that a) most of the mutations in the beginning of the genes in the wild-type strain occur in the non-transcribed strand; and b) the absence of *sub1* causes a strong increase in deaminations in the transcribed strand in TSS/5’-UTRs, while exerting only little effect on the non-transcribed strand deaminations.

In order to better understand the nature of the changes in mutation distribution in *sub1* strains, we have estimated the strand specificity of mutations for the different genomic features (Fig 4). In the wild-type strain, the strongest strand specificity was observed in the 5’-UTRs, then in pre-5’-UTR regions, and only a slight bias was found in the coding sequences. Disruption of *sub1* suppressed strand specificity in all features. Strand bias was observed only in 5’-UTRs, and it was lower than in the wild-type. These results are in perfect agreement to the strand-specific effects illustrated in Fig 10.

## Discussion

Understanding the mechanisms of mutagenesis is fundamental for the theory of evolution, including the evolution of tumors. For a long time, mutations were considered to occur in a random fashion. In the last few decades, evidence has accumulated for the existence of mutation clustering (“too many mutants with multiple mutations”, following the Drake’s pun) [36–39]. Recent discoveries of clustered mutations in cancers added a new dimension to the mutagenesis research (reviewed in [40, 41]). Mutational clusters are clearly found in the genomes of various tumors and model organisms. For research purposes, it is important to develop the tools for the detection and classification of these clusters. Recently, a method of cluster detection based on negative binomial distribution was developed and applied to both yeast and human cancers mutational data [42]. One goal of our study was to expand the current arsenal of cluster detection methods [42–44] by the new approach, that robustly detect clustering in relatively small but heavily mutagenized yeast genomes when mutation density is very high.

We have developed a new method of mutation clustering detection (see Methods for the overview). By applying this method to the dataset of SNVs from PmCDA1-induced mutants, we were able to detect multiple and diverse clusters. We set up a cophenetic distance threshold to 1,000 bp to prevent combining of small clusters into larger ones by the algorithm (more details in Results section). Each cluster is characterized by size and power (see Materials and Methods). Results of clustering analysis (Fig 3) are in a good correlation with the rainfall plots, which allow for easy visualization of mutation clustering (Figs 2 and S1). Application of this method to the SNV data from yeast mutagenized with chemical mutagen, 6-HAP, provides a means to estimate the degree of background clustering. We observed weak clusters in HAP mutation data (Fig 3). It is possible that there are a few sites in the genome that are more susceptible to HAP-induced mutations.

In the *ung1* strains, almost all mutations represent direct deamination of the corresponding nucleotide (28). Therefore, the nature of substitution (C->T or G->A) indicates which one of the complementary DNA strands was deaminated. We have calculated the homogeneity of clusters by determining the major type of substitutions within the cluster, and then calculating the percent of non-major SNVs within the same cluster. Clusters with various homogeneity have been observed (Fig 3E and 3F). In addition to completely polar clusters (homogeneity = 1, consists of only one type of substitutions—either C->T or G->A), there were “mixed” clusters with intermediate homogeneity. Polar clusters are likely to represent the deaminations of ssDNA formed in the one event of dsDNA unwinding, such as transcription of a particular gene, or replication intermediate/pause site, or recombination event. We found a highly significant strand bias toward C->T mutations in the genes located in the Watson strand of the genome, and toward G->A mutations in the genes located in the Crick strand (Fig 4). Strikingly, genes with higher expression level were enriched with mutations (Figs 8 and S5). Taken together with the overall setup of our experimental system, where cells are not dividing most of the time, these data strongly support the hypothesis that APOBEC preferentially deaminates the non-template DNA strand during transcription. However, the strand specificity was not absolute and, interestingly, it also varied across the different genome features (Fig 4). The strongest strand specificity was observed in the 5’-UTR regions, and then it progressively decreased in the pre-UTR regions, followed by coding sequences; no strand-specificity was observed in the 3’-UTRs (Fig 4). Moreover, a strong peak of mutations was found in the beginning of the genes, right before the start codon (Fig 7). This was true for all deaminases examined, including the human AID, APOBEC1 and APOBEC3G proteins (S3 Fig), even though they tend to target different sequence motifs (see the results on Fig 1, which is in agreement with [19, 28]). This finding explains the observed strong bias toward the non-random accumulation of deaminase-induced mutations in the genomic regions annotated either as 5’-UTR or “intergenic” (compare results for PmCDA1 and 6-HAP in Fig 5). Indeed, higher mutation densities have been found in the intergenic regions in human cancer genomes; and some of the mutations affected transcription factor binding [45]. In another study, the peak of strand-coordinated C->T/G->A mutations was detected in the 1–2 Kb regions downstream of TSS in the human genome [46]. On the other hand, recent analysis discovered a negative correlation between the expression level and mutation density in cancer genomes [47]. On the evolutionary scale, the general rule is that highly expressed genes are changing slowly (reviewed in [48, 49]). Transcription is well-known to cause mutagenesis and genome instability (reviewed in [50, 51]). These apparent contradictions may represent the heterogeneity of samples that were used for the analysis. It is likely that the positive correlation between the expression level and mutation load will be observed if deaminases played a significant role in shaping the particular genome(s). On the other hand, in the other genomes, the correlation will be negative due to the efficiency of transcription-coupled repair [52]. Transcription is known to play a significant role in deaminase targeting during somatic hypermutation (or other processes) [53–61]. Our data elaborate this model and suggest that the non-template strand is especially prone to deamination in the region of promoters and transcription start sites. Recently, similar transcription start site effects on deaminase targeting yeast genomic DNA were detected [62]. Interestingly, both single- (PmCDA1, AID, APOBEC1) and double-domain (APOBEC3G) APOBEC proteins were inducing mutations in the beginning of the genes (Figs 7 and S3), indicating that protein size is not a major determinant in deaminase targeting. This is in agreement with the data from [62] where two-domain APOBEC3B induced similar peaks of mutations.

Based on our results and explanations, we can propose several mechanisms for the formation of mixed clusters (0.5 = <homogeneity<1). First, two closely located, or even overlapping (if the corresponding genes are located in different DNA strands), polar clusters of different mutation type can be classified by the algorithm as one cluster. Two examples of this type of clusters are shown in Fig 6A (non-polar cluster overlap with both *GAL10* and *GAL1* genes sharing a bi-directional promoter), and Fig 6C (intermediate polar cluster involving different genes). From the standpoint of genetics, it is indeed one cluster (with the correction to the data pooling, see Materials and Methods), but mechanistically it is formed by the two (or more) virtually independent processes, such as transcription of neighboring genes. Second, it is possible that deaminase can also access DNA during the replication, although with much less probability than during transcription. This will lead to another layer of SNVs which will not be correlated to the gene orientation. The combination of “replicative” and “transcriptional” sets of mutations will result in mixed clusters. Third, it is also possible that the transcribed DNA strand can be also accessed by the deaminase. The non-transcribed strand is thought to be better protected by the polymerase complex and accessory proteins [63–65]; however, the precise contribution of multiple factors is not known. Plotting the strand-specific mutation densities revealed that the peak of mutations at the beginning of the genes in the wild-type (*SUB1*) strain is formed primarily by the non-transcribed strand deaminations (Fig 10, left panel of plots). However, some non-transcribed strand deaminations are clearly present (Fig 10, third row of plots, left side). Recently, it has been shown that ssDNA binding protein RPA, which is well-known for its roles in replication, repair and recombination [66, 67], is associated with expressed genes [35]. In this model, RPA binds the non-template DNA strand during transcription, protects it from damage and stimulates the RNA polymerase progression. RPA has been shown to protect ssDNA from deaminations by APOBECs [21–23]. However, in the regions of transcription initiation, RPA is substituted for the Sub1 protein [35]. RPA and Sub1 use similar modes of ssDNA binding (through the “half-pipe” and “quarter-pipe”, respectively; [68, 69]). Sub1 is known as a transcription initiation factor [70], however, its precise role in the overall process is unknown. We hypothesized that Sub1 protects ssDNA from deaminases less efficiently than RPA, and it can explain the observed peak of mutations in the promoter/5’-UTR regions.

In the strains with a disrupted *SUB1* gene, we found that the peak of mutations in the -100:0 bp window relative to CDS start is ~2x stronger as compared to the wild-type (Fig 7). Moreover, there was a second peak directly upstream from the first one. While the first peak corresponds to the TSS in both *sub1* and wild-type strains, the second peak was found in the pre-5’-UTR region, thus representing deamination in the promoter. Additionally, there is a two-fold decrease in SNV density in the CDS regions in the *sub1* strain (Fig 7). No such effects were observed in the 6-HAP data (Fig 7). The re-distribution of APOBEC-induced mutations suggests that the probability of deamination in the beginning of the genes in the *sub1* strains is increased. This is achieved at the cost of decreased deaminations in the coding sequences, likely because the concentration of active APOBECs in the cells is limited. This model explains the reduction in PmCDA1-induced mutation frequency (Can^R^) in the *sub1* strain (Fig 9), and the seemingly paradoxical observation that the Can^R^ mutants obtained in the wild-type and *sub1* strains possess similar numbers of SNVs per genome, even though the observed hundred-fold decrease in mutation frequency in the *sub1* strain. The expression profiles from *SUB1* and *sub1* cells were virtually identical (S4 Fig), but the expression values (FPKM) obtained from the RNA-Seq data are normalized for the total RNA content and cannot be used to estimate overall expression changes. It has been shown that the *sub1* mutation in yeast reduces levels of transcription [35]. The combined effect of mutation densities re-distribution and the overall transcription decrease helps to explain the strong reduction of the phenotypically detected mutants in the *sub1* strain.

Importantly, the effects of other mutagens were not modified by Sub1 inactivation (Figs 9 and S6). The *sub1* deletion yeast mutant has been shown to exhibit a slight elevation in spontaneous and peroxide-induced mutagenesis [71]. The difference between this work and spontaneous mutagenesis data are most likely explained by the semi-qualitative nature of spot-test assay, which does not always allow for the detection of small differences in mutagenesis rates. Nevertheless, it is striking that *SUB1* inactivation leads to strong anti-APOBEC mutagenesis in a reporter gene and a slight increase in spontaneous and oxidative stress-induced mutagenesis [71].

Strand bias of deamination was highly suppressed in the *sub1* strain (Fig 4). In fact, significant strand specificity was observed only in the 5’-UTRs, and it was lower than in the wild-type. Moreover, strand-specific plots of mutations revealed that the increase of the promoter/5’-UTR mutation peak in *sub1*-defective strains is mediated by the deaminations of the transcribed strand (Fig 10, compare second and third rows of plots). Therefore, it is likely that Sub1 is protecting from APOBECs the transcribed strand in the beginning of the genes. The easiest explanation for this finding is that Sub1 is associated with the transcribed strand during the initiation; then loss of Sub1 will expose the DNA and lead to a decrease in strand bias. However, alternative explanations are also possible. First, Sub1 may be bound to both strands of the DNA in the promoter and transcription initiation regions. The non-transcribed strand is still less protected from the damage, therefore there is a peak of strand-biased mutations in the wild-type strain. Without Sub1, both strands become more accessible for deaminase, thus contributing to the decrease in strand specificity. Second, it was shown that, in the absence of Sub1, RPA is bound to the initiation regions [35]. If it only binds to the non-transcribed strand it will be better protected. At the same time, the initiation may become slower without Sub1, thus leading to an overall increase in mutagenesis in this region due to prolonged exposure of the promoter in the ssDNA form. Another possibility is that access of RPA to the regions of initiation is limited or strand-specific, and it cannot fully compensate Sub1 in the ssDNA protection. Finally, it is possible that the absence of Sub1 increases supercoiling during transcription, which is known to cause targeting of the deaminase to both DNA strands [72]. This process can be linked to the formation of R-loops [73, 74] that are known to be the target of APOBEC [75]. PC4, human Sub1 ortholog, is involved in replication, DNA repair and transcription (reviewed in [70]). It will be interesting to check whether the knockdown of PC4 will lead to an increase of localized APOBEC-induced DNA damage. Additional studies are required to better understand the molecular mechanisms of co-transcriptional Sub1-RPA-deaminase physical and functional interactions.

In our model system, diploid eukaryotic cells undergo genome-wide mutagenesis after treatment with either enzymatic (APOBEC), or chemical (6-HAP) mutagens. As a result, some cells acquire resistance to the drugs used to select mutants (canavanine or 5-FOA). In diploids, both alleles of the reporter genes (*CAN1* or *URA3*) need to be inactivated to produce a detectable phenotype. We have previously shown that mutagenized unselected clones have lower mutation loads in genomes. In the case of PmCDA1, these non-mutant clones accumulated just a few mutations per genome [28]. This underscores the importance of the selection in the success of the recovery of multiple mutations and their clusters. Some cells are more prone to the deaminase-induced mutagenesis due to natural variability in cellular parameters, such as levels of production and degradation of APOBEC itself and ssDNA-protecting proteins (e.g. RPA, Sub1, and Rad51) and their post-translational modifications and intracellular localization. The other contributors are overall transcription levels, chromatin states, and the physiological/biochemical environment in the cells. The progeny of such cells gives rise to the Can^R^ and FOA^R^ mutant clones, which are then selected and analyzed by NGS. Similarly, cancer development is an evolutionary process driven by selection of the fittest clones (reviewed in [76, 77]). The selection during cancer progression is fundamental for tumor growth, since cancerous cells need to overcome multiple barriers in order to keep proliferating and conquer the host body. Selection occurs during an early evolution of tumors and also in metastasis. It also mediates the acquisition of drug-resistance after chemotherapy. It is possible that APOBECs can participate in the shaping of the cancer genome at all of these stages. Our yeast system models some aspects of the APOBEC-dependent stages of cancer evolution.

**Fig 11:**
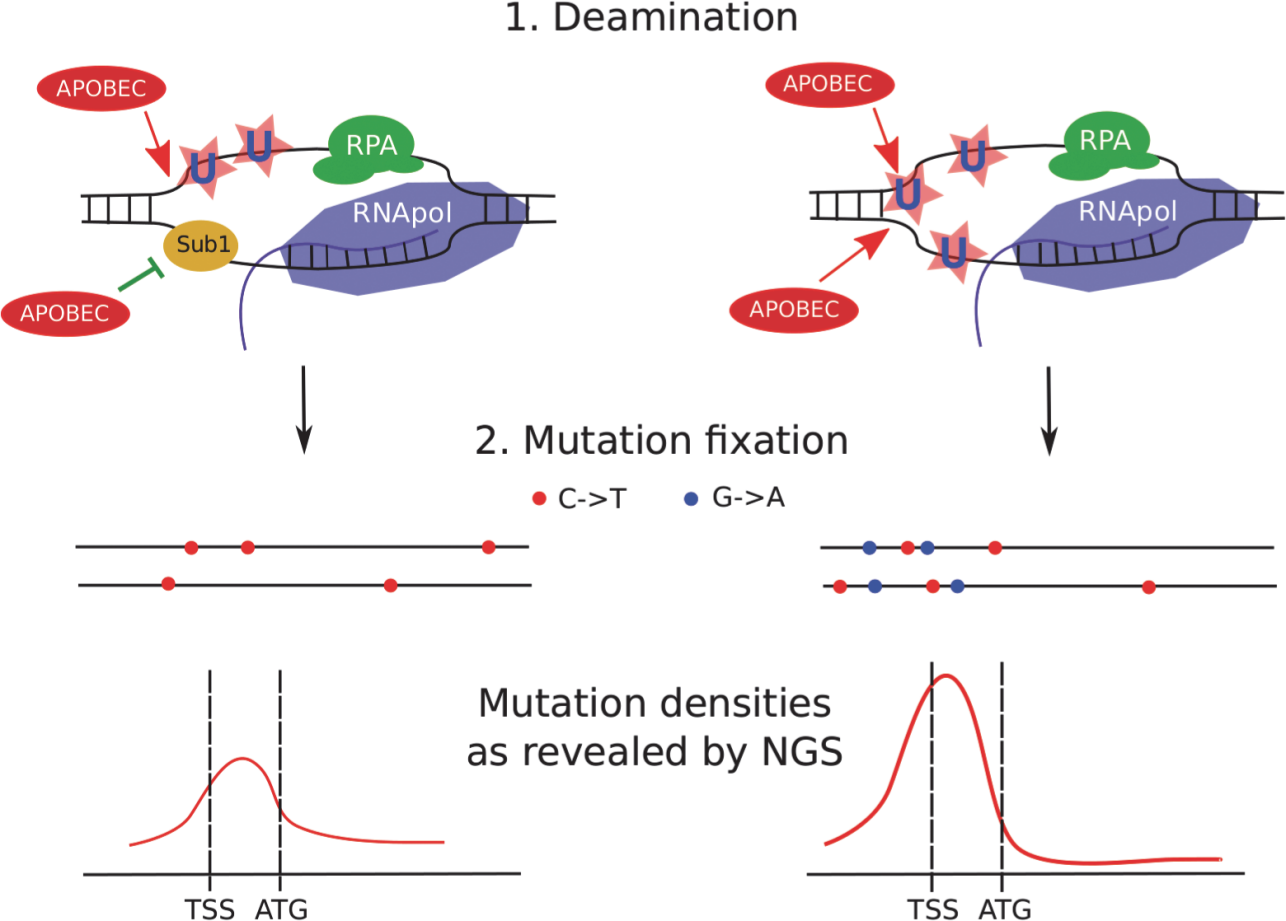
Model of transcription-dependent induction of clustered mutations in diploid cells. Left, *SUB1* (“wild-type”) strain. Regions of ssDNA formed during initiation of the transcription are particularly vulnerable to APOBEC which deaminates the regions of transcription initiation/early elongation, leading to uracil (U) in DNA. The non-template strand is excluded from the polymerase complex. Sub1 provides a certain level of protection of promoter/TSS DNA. It is unknown whether it is bound to one or both DNA strands, although the simplest interpretation of our data is that it is bound only to the transcribed strand (also see Discussion). Gene bodies are better protected from deamination by RNA polymerase and RPA on the template and non-template strands, respectively. Fixation of mutation leads to the formation of heterozygous, predominantly C->T mutations (red dots) in both chromosomes (horizontal black lines) of the diploid cell with mutational peak around the promoter/5’-UTR region. (For simplicity, only the genes in the Watson strand of the genome are shown). Right, *sub1* strain. Loss of protection factors, such as Sub1, enhances the effect of promoter/TSS targeting, leading to stronger clusters of heterozygous mutations in the beginning of the active genes. Because both strands become accessible to APOBEC, C->T and G->A mutations accumulate and the mutation density peak becomes stronger at the cost of reduced mutagenesis in protein-coding regions.

Based on these and other results, we propose the following model of generation of mutant clones in the diploid cells (see Fig 11). Clustered mutations are generated genome-wide or locally in the presence of ssDNA-specific mutagen, such as deaminase. For example, transcription-coupled deamination in the beginning of the genes will create clusters that will unlikely directly affect the protein product, but may change gene expression and its regulation. The parameters of mutational clusters depend on many factors, including the level of protection of ssDNA in the nuclei. Transient loss of protection factors, such as Sub1 and RPA proteins, lead to the formation of stronger clusters. Resulting clusters of heterozygous mutations can either directly change the phenotype in case of a dominant negative effect, or require an additional loss of heterozygosity (LOH) step in order to create a homozygous cluster. All of these processes—deamination, loss of protection, and LOH—act synergistically to shape the genome of the mutant cell, including tumor clones.

To conclude, APOBEC deaminases induce clustered mutations in diploid yeast cells by accessing ssDNA in the active genes. Regions of promoters and transcription initiation are preferentially targeted by the enzyme. Loss of the Sub1 protein further increases this effect, leading to genome-wide redistribution of mutation densities. Unexpectedly, in the case of *sub1* mutant strains, there is no correlation between mutation frequency measured by methods of classical genetics and next-generation sequencing. Loss of ssDNA-binding proteins, such as Sub1 and RPA, on the background of APOBEC expression, can explain cancer initiation and progression, including *kataegis* (see the model presented in Fig 11).

## Materials and Methods

### Yeast strains and plasmids

Strains used in this work are described in [28]. To construct *sub1*-deficient LAN210 derivative, we PCR-amplified the *sub1*::*KanMX* cassette from BY4742 *sub1*::*KanMX* strain, and used the resulting PCR product to transform the LAN200 strain (haploid *ung1*, [23]; genotype *MAT*a *ade5-1 lys2-Tn5-13 trp1-289 his7-2 leu2-3*,*112 ung1*::*hygB*). We PCR-verified the resulting Kan^R^ clones and transformed them with plasmid YEpHO encoding for HO endonuclease. Autodiploids (LAN210 *sub1*) have been selected and used for mutagenesis experiments.

### Mutagenesis experiments

Yeast strains LAN210 or LAN210 *sub1* were transformed with plasmids pESC-*LEU*-PmCDA1, pESC-*LEU*-AID, pESC-*LEU*-APOBEC3G, or pESC-*LEU*-APOBEC1 [27], and transformants were colony-purified and inoculated into 5 ml of synthetic complete medium (SC) lacking leucine and containing 2% glucose. After one day of incubation, cultures were spun down, washed with water and resuspended in 12 ml of SC containing 1% raffinose and 2% galactose. After three days of culture growth, aliquots were plated on SC plates with 60 mg/L of L-canavanine to select for *can1* mutants and to the synthetic complete plates (with dilutions) to estimate viability. Some mutants were selected on 5-fluoroorotic acid (5-FOA) plates (0.1% 5-FOA) instead of canavanine plates. Clones resistant to either canavanine or 5-FOA were selected, colony purified and stored at -80C.

Our original tests indicated that glucose-containing medium during the first stages of culture growth can be substituted for the synthetic medium lacking glucose but with raffinose. Later, we found that the mutation frequency can vary under these conditions. We do not know the exact reason for this high variability under raffinose pre-growth conditions.

In addition to the forward mutation assay (*CAN1* gene) we have analyzed the reversions on *his7-2*, *trp1-289*, *lys2-Tn5-13*, and *ade5-1* mutant alleles [27] in the spot test (S6 Fig). Yeast of the corresponding strains were streaked on YPDU plates. One set of plates was UV-irradiated (20J/m^2^), another set was HAP-mutagenized as described in [28], and the third was left untreated to assay for spontaneous mutagenesis. After one day, yeast cells were replica-plated only onto corresponding selective plates. Colonies were scored after three days of growth.

### Preparation of libraries for next generation sequencing

Genomic DNA was prepared as described in [28]. We used two methods of library preparation, both from Illumina: TruSeq was used for majority of the clones (containing “RUN1” to “RUN5” in systematic names of clones, see Table 1), and Nextera XT (those clones that contain “RUN6” in the clone name). All libraries have been sequenced by 100 bp paired-end reads, except for the clones containing “RUN4” in the name, which have been sequenced using 100 bp single-end reads. Raw sequencing reads (fastq format) have been deposited to the Sequence Read Archive (SRA), accession numbers SRP056337 (whole genome sequencing files), and SRP056371 (RNA-seq data).

To improve the quality of our reference genome assembly, we have sequenced wild-type strain LAN200 on the Roche Junior instrument (Research and Resource Center “Molecular and Cellular Technologies”, Saint Petersburg State University, Russia) using 454 technology according to the manufacturer’s recommendations.

RNA was purified using a RiboPure Yeast Kit (Life Technologies, Ambion, cat. number AM1926). To prepare the libraries for RNA sequencing, we used the ScriptSeq Complete Gold Kit (Yeast) (Epicentre cat. number BGY1306). This kit allows for effective depletion of all yeast rRNA species and also preserves information about RNA strand. Size distribution of the resulting libraries was estimated by both agarose gel electrophoresis followed by SYBR Gold or SYBR Green (both from Life Technologies) staining and visualization on Typhoon scanner, and Bioanalyzer (Agilent Technologies). We have used two RNA samples from the LAN210 strain expressing PmCDA1, and one RNA sample from the LAN210 *sub1* strain expressing PmCDA1. RNA was purified from the same cultures used to measure mutation frequencies. The libraries were pooled together and sequenced on a HiSeq2500 instrument using 100 bp paired-end reads.

### Filtering and error correction of next generation sequencing data

All DNA Illumina reads were filtered by base quality (threshold Phred score 20), 5' trimmed (see S1 Protocol for details). Adapters were filtered from the 3' end of reads (S1 Protocol). Minimum read length after filtering was set to 20. The filtering steps were performed using Trim Galore! (http://www.bioinformatics.babraham.ac.uk/projects/trim_galore/). Next, filtered reads were corrected by BayesHammer module of Spades assembler [78].

Roche reads were cut to a length of 479 and then 11 bp from the 5' end of reads were trimmed. After this, reads were filtered by base quality using fastq_quality_filter (parameters-q 20-p 80) from FastX package (http://hannonlab.cshl.edu/fastx_toolkit/index.html).

### Assembly of LAN210 reference genome

We have improved the genome of our reference strain LAN210 [28] by taking advantage of longer reads obtained from Roche sequencing run. Assembling and correction of assembling errors were performed in several steps (see diagram in S7 Fig).

In the first step, N008-LAN210-wt-NA-NA-RUN2 and Roche reads were assembled *de novo* to contigs using Mira assembler v 4.0 (http://www.chevreux.org/projects_mira.html). Contigs with a length of 500 bp and more were aligned to standard yeast reference genome S288C_R64 using BWA (mem algorithm) 0.7.5a (http://bio-bwa.sourceforge.net/). Then, the consensus sequence (designated as “raw reference”) was extracted from alignment using SAMtools v0.1.19 ([79], http://samtools.sourceforge.net/).

In the second step, filtered and corrected sequencing reads from the wild-type LAN210 strain (N008-LAN210-wt-NA-NA-RUN2) were aligned to the “raw reference”, and variants were extracted from alignment as described in the section **Detection of variants**. Then homozygous variants were used to correct the “raw reference” to the “intermediate reference” using GATK (https://www.broadinstitute.org/gatk/).

In the last step, common homozygous variants from alignments of corrected and filtered reads from sequencing LAN200 and LAN201 strains (clones N001-LAN200-wt-NA-NA-RUN1 and N002-LAN201-wt-NA-NA-RUN1) were used in the same way as described previously to get the final reference genome (S2 Dataset—LAN210_v0.10m genome in FASTA format).

### Annotation of LAN210 reference genome

The Saccharomyces Genome Database (SGD, www.yeastgenome.org) lacks UTR annotations. We have combined the published UTR annotations [34] with SGD annotations of standard yeast reference S288C (S288C_R64). These merged annotations were transferred to the LAN210_v0.10m genome sequence using a custom script based on BLAST. The annotations in the GFF format can be found in S3 Dataset.

### Repeat masking

Repeats in LAN210_v0.10m were masked using four sources (see S1 Protocol and S7 Fig). First, interspersed repeats were discovered *de novo* using Repeat Modeler (http://www.repeatmasker.org/RepeatModeler.html). Then, they were combined with known fungal repeats from RepBase (http://www.girinst.org/repbase/index.html), and both were masked using Repeat Masker (http://www.repeatmasker.org/). Second, tandem repeats were raw masked using TRF ([80], http://tandem.bu.edu/trf/trf.html). Then, raw masked tandem repeats were checked for over masking (known problem of TRF) using LAN210_v0.10m annotations: entries in genes were removed. Third, all tRNA features were masked. Fourth, manual masking based on BWA alignment of contigs (see **Assembly of LAN210 reference genome**section) to LAN210_v0.10m was performed, resulting in regions with conflicting contigs being masked.

### Detection of variants

For each clone/sample (see Table 1), corrected and filtered reads (as described previously) were aligned to the LAN210_v0.10m genome using Bowtie2 v2.1.0 ([81], http://bowtie-bio.sourceforge.net/bowtie2/index.shtml) in “very-sensitive” mode. Then, the variant call was performed using Unified Genotyper (with options-stand_call_conf 100-stand_emit_conf 40) from GATK, for both indels and SNPs. After that, SNPs and indels were split and independently filtered according to the GATK's Best Practices (https://www.broadinstitute.org/gatk/guide/best-practices). Finally, filtered indels and SNVs from LAN200, LAN201, and LAN210 (N008-LAN210-wt-NA-NA-RUN2, N001-LAN200-wt-NA-NA-RUN1, and N002-LAN201-wt-NA-NA-RUN1) were combined. Only SNVs were further considered for all other samples. For SNVs in deaminase-induced mutants, the last filtration step was associated with clustering (described below).

### Analysis of mutation clustering

Genomes of diploid yeast mutants induced by deaminases possess hundreds to thousands of mutations (]28] and Table 1), making these data a good material to detect the hypermutable genomic regions. To increase the sensitivity of detection even further, we pooled the mutations from the clones induced by the same deaminase together. The resulting datasets are characterized by very high mutation densities over the relatively small yeast genome. because of that, we developed a sophisticated clustering procedure that was applied for combined SNV sample sets and consisted of several stages (see scheme in S8 Fig).

**In the first step**, hierarchical clustering was performed using the SciPy library (http://www.scipy.org/). In hierarchical clustering, objects are grouped together (based on distances between them) in nested clusters until a single cluster harboring all objects is formed. The structure of derived clustering can be easily represented as a tree. At this time, the choice of a method to calculate the distances between objects and clusters is highly important. However, there is still no general approach to make such choice. So, one has to choose an appropriate method based on the features of the data he is studying. We choose an average distance (UPGMA) method to calculate the distances between clusters (the reasons are described below). In this case, a distance *d(u*, *v)* between two clusters of objects *u* and *v* is calculated as follows:

$$d\left(u,v\right)\;=\;\sum_{ij}\frac{d(u\left[i\right],\;v\left[j\right])}{\left(\left|u\right|*\left|v\right|\right)}$$

where *d(u[i]*, *v[j])* is the distance between the *i-th* object from cluster *u* and the *j-th* object from cluster *v*, and |*u*| and |*v*| are numbers of objects in clusters *u* and *v*, respectively. The average distance used to perform hierarchical clustering and the distance criterion used to extract clusters do not set strict limitations for cluster length. Importantly, 1 Kb is not the maximum length of a cluster, as some clusters are longer; the threshold is based on cophenetic distance (see below). Also, the UPGMA method takes into account distances between all mutations in the cluster in comparison to NPA (nearest point algorithm) or FPA (farthest point algorithm).

If there is no interest in hierarchical clustering process as it but instead one would like to obtain a set of clusters, a method and threshold to extract them from the clustering “tree” have to be chosen. We choose a distance criterion as the most appropriate method. This means that all SNVs in each extracted cluster should have no greater than a cophenetic distance than the threshold. The cophenetic distance between two objects is the distance between the largest two clusters containing these objects individually when they are merged into a single cluster that contains both. In other words, it is the height of the tree where the two branches, including the two objects, merge together. For our purposes, “clusters” are defined as “clusters extracted from hierarchical clustering”. It is important to distinguish them from nested clusters of hierarchical clustering.

To set a threshold for extraction of clusters, we performed an extraction with different values of threshold in a range from 50 bp to 5000 bp with a 50 bp step. The number of clusters with three or more mutations reaches or nearly reaches a plateau when the threshold was set to a value in the range of 1000 to 2000 bp (S2 Fig). Considering the high density of mutations in our dataset, and *a priori* knowledge of approximate cluster size (rainfall plot) we have set the cutoff for extraction to 1000 bp.

The distance criterion was used for extraction because, except for clustered mutations, there was a “noise” consisting of “random” mutations. It was impossible to predict the number of clusters or to directly use more “natural' methods, such as an inconsistency coefficient (which was used to adjust clusters in the next step).

**In the second step**, hierarchical clustering was performed independently for the SNVs in each cluster, and subclusters were extracted using inconsistency coefficient with the threshold set to 0.8 and depth set to two to adjust the borders of clusters. The inconsistency coefficient characterizes each link in a cluster tree by comparing its height with the average height of adjacent links that are less than the depth value below it in the cluster hierarchy. The higher value of this coefficient indicates the lower similarity of the objects connected by the link.

**In the third step**, a filtering of clusters was performed. All clusters with one or more SNV in the masked (see **Repeat Masking**above) regions were removed. Also, for deaminase sample sets, clusters with more than one non-deaminase-like SNV (non- C->T or G->A) were considered as errors of sequencing or SNV call and removed from analysis. In other words, filtering of SNVs for deaminase sample sets is merged with clustering filtration. Clusters with one non-deaminase-like SNV present in only one sample in a set were marked and corresponding SNVs were removed. At this step, SNVs for each sample were independently restored from clustering results. The final SNV datasets can be found in S1 Dataset.

Finally, clusters were filtered based on their size (only clusters with five and more SNVs were retained) and power (calculated as the size of a cluster divided by the median distance between SNVs in the cluster). The minimum power threshold was set to 0.05 to remove the clusters with a low density of SNV that may be due to high-level “noise” because of high mutation densities.

### RNA sequencing data analysis

We have used Top Hat followed by cufflinks [82] in annotation-guided modes to obtain FPKM values for the genes annotated in our reference genome. FPKM stands for fragments per kilobase per million mapped reads and represents relative expression values for the genes and/or transcripts.

### Other bioinformatics techniques

Analysis of mutation densities around CDS start/UTR/TSS was performed using custom scripts (S2 Protocol). Sequence logos (Fig 1) were created from pooled SNV data in WebLogo 3.0 (http://weblogo.berkeley.edu/), using *S*. *cerevisiae* GC content (38%) for values normalization. Sequences were reverse-complemented where applicable using FastX-Toolkit (http://hannonlab.cshl.edu/fastx_toolkit/). Due to the relatively low number of mutations in the genomes of APOBEC3G-induced mutants and the resulting increased ratio of false positive SNVs to the real mutations, the results of the automatic SNV call were manually checked in the genome browser. The manually filtered SNVs dataset was used for logo creation.

### Statistics

Most of the statistical tests have been computed in R.

## Supporting Information

S1 Dataset SNV dataset. (ZIP)

S2 Dataset Sequence of LAN210 reference genome, version LAN210_v0.10m. (FASTA)

S3 Dataset Annotations for the LAN210_v0.10m reference genome, including UTRs. (GFF3)

S1 Fig Rainfall plots for data on 6-HAP- and PmCDA1-induced mutations. (PDF)

S2 Fig Dependence of the number of detected clusters on the cophenetic distance threshold. Results for 6-HAP and PmCDA1 data for the clusters with different sizes (number of SNVs) are shown.
(PDF)

S3 Fig Mutational densities around CDS start for the APOBEC1, AID, and APOBEC3G. (TIF)

S4 Fig Comparison of expression profiles in RNA-Seq samples. Name explanation: wt_cl1 means wild-type (*SUB1*) clone 1.
(PDF)

S5 Fig Expression levels of the genes with different numbers of mutations. Y-axis, log_10_(FKPM) values. Other elements of the graph are as described in the legend to Fig 8.
(TIF)

S6 Fig Comparison of spontaneous, 6-HAP-, and UV-induced mutagenesis in wild-type and *sub1* strains using spot-test. First page. Location of patches with different strains is shown on the scheme at the right of the page. SC-His, synthetic complete media without histidine; SC-Arg+CAN, synthetic complete media without arginine and containing canavanine. Second page. Results on synthetic complete media plates without adenine, tryptophan and lysine (SC-Ade, SC-Trp, and SC-Lys, respectively). The pattern of strain patches is the same as on page 1.
(PDF)

S7 Fig Scheme of LAN210 reference genome assembly, annotation, and masking. (PDF)

S8 Fig Scheme of SNV call and mutation clustering analysis. (PDF)

S1 Protocol Details of processing of raw sequencing data. (INFO)

S2 Protocol Scripts used to analyze mutation densities in the beginning of genes. (ZIP)
