# Supplementary figures and images for: Disruption of Transcriptional Coactivator Sub1 Leads to Genome-Wide Re-distribution of Clustered Mutations Induced by APOBEC in Active Yeast Genes
Source: PLoS Genet. 2015 May 5;11(5):e1005217. doi: 10.1371/journal.pgen.1005217 (PMC4420506; doi:10.1371/journal.pgen.1005217)

HAP

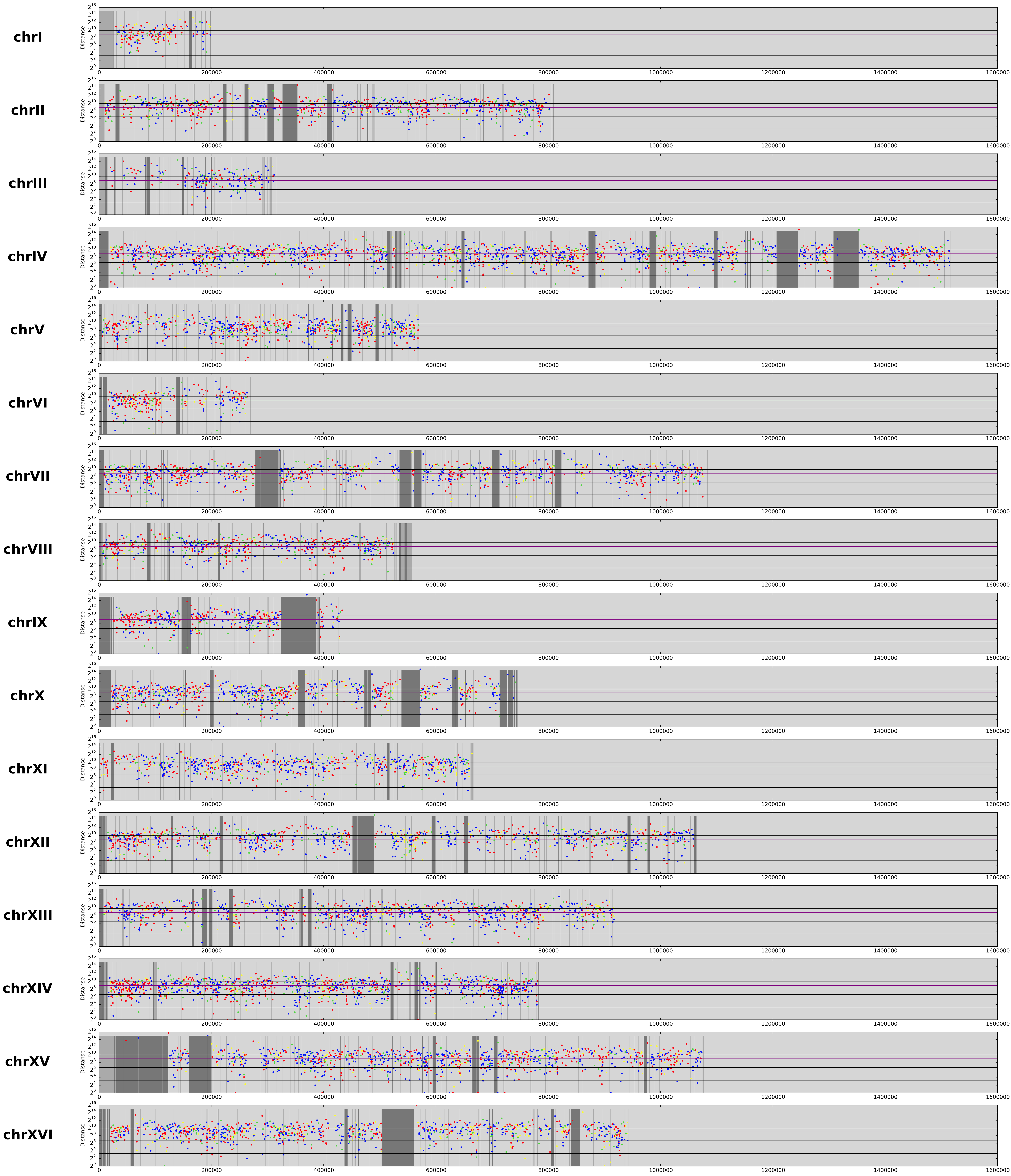

**HAP\_sub1**

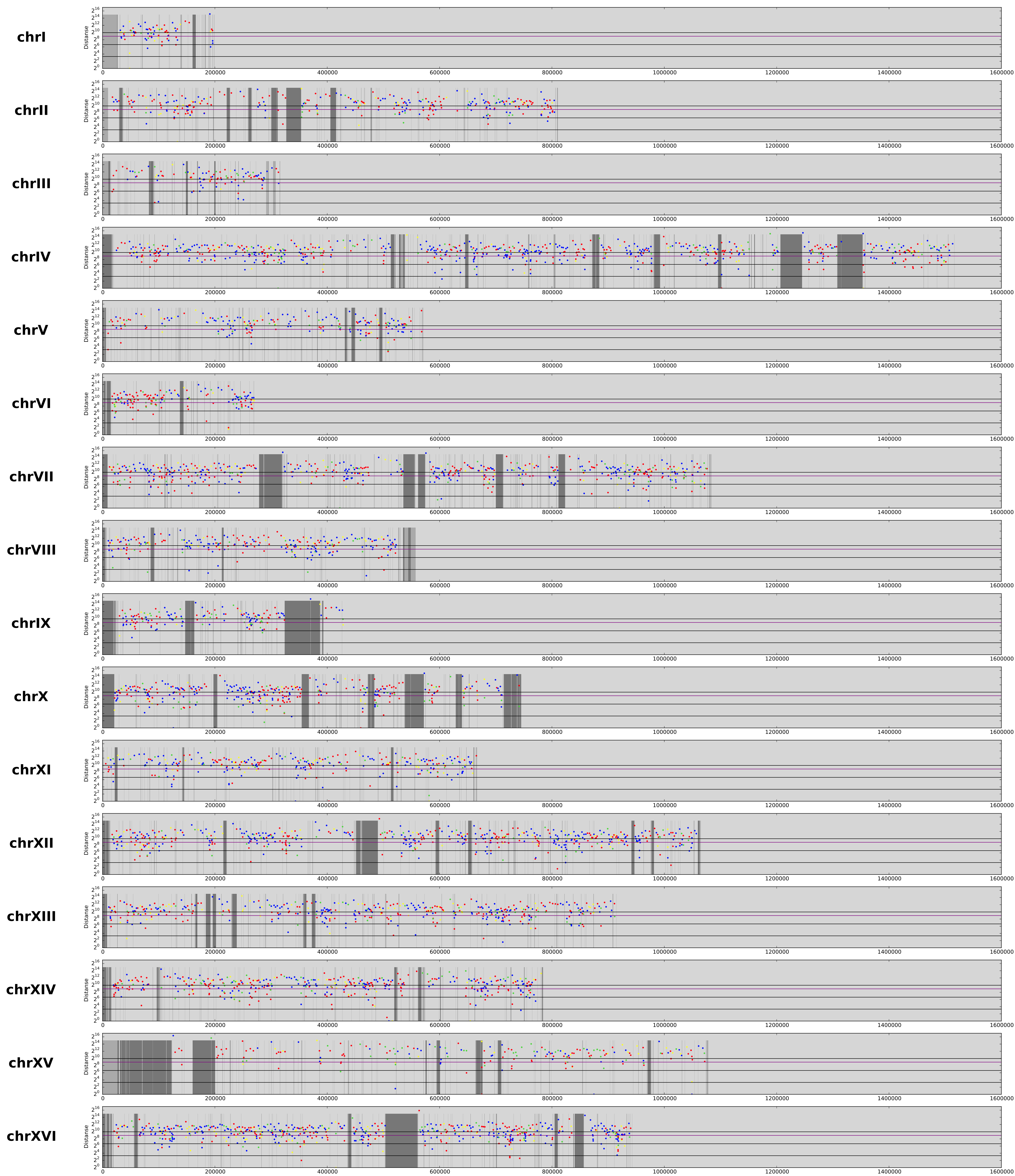

## PmCDA1

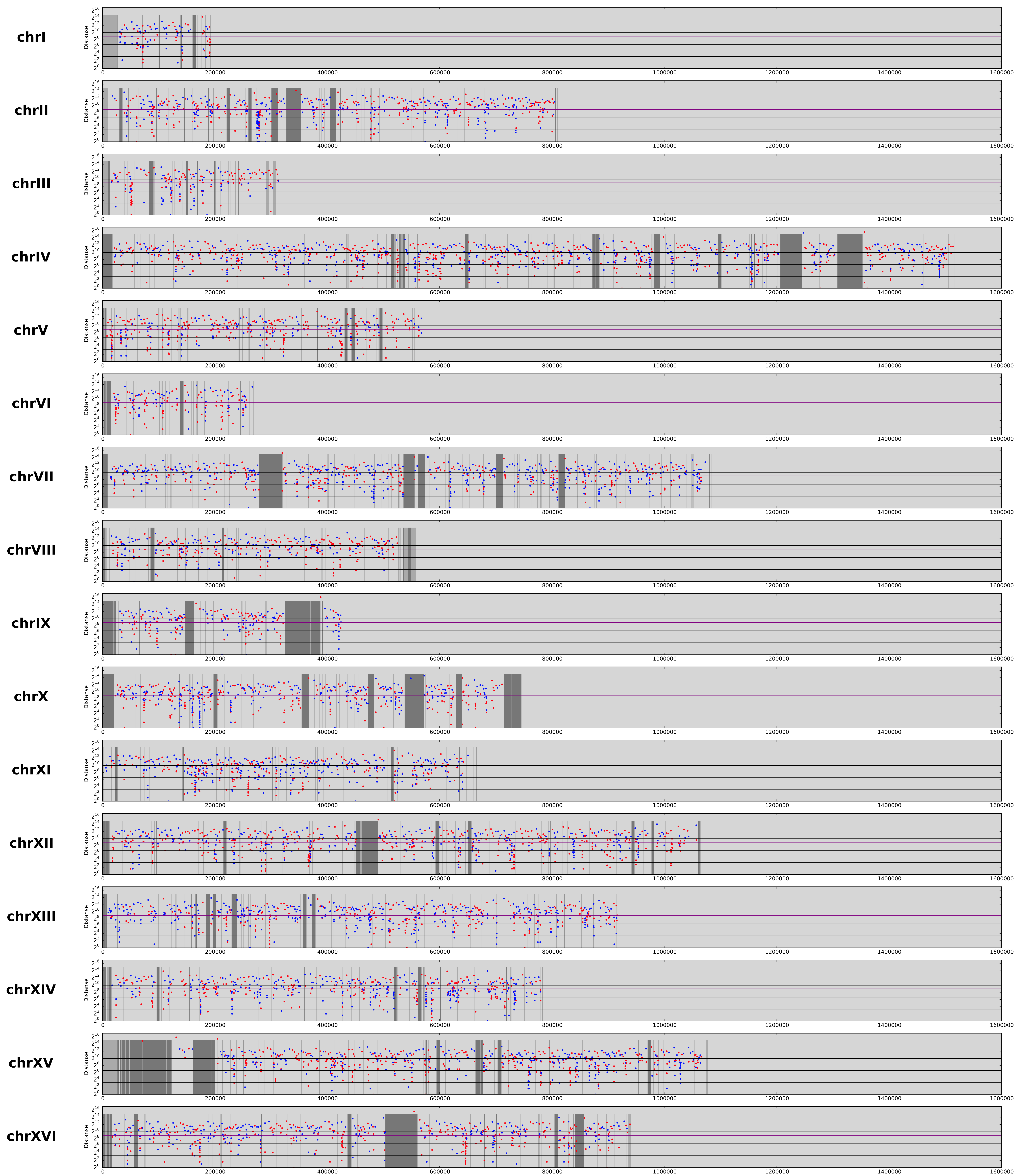

**PmCDA1\_sub1**

Supplement: S1 Fig — (PDF) [file pgen.1005217.s004.pdf]

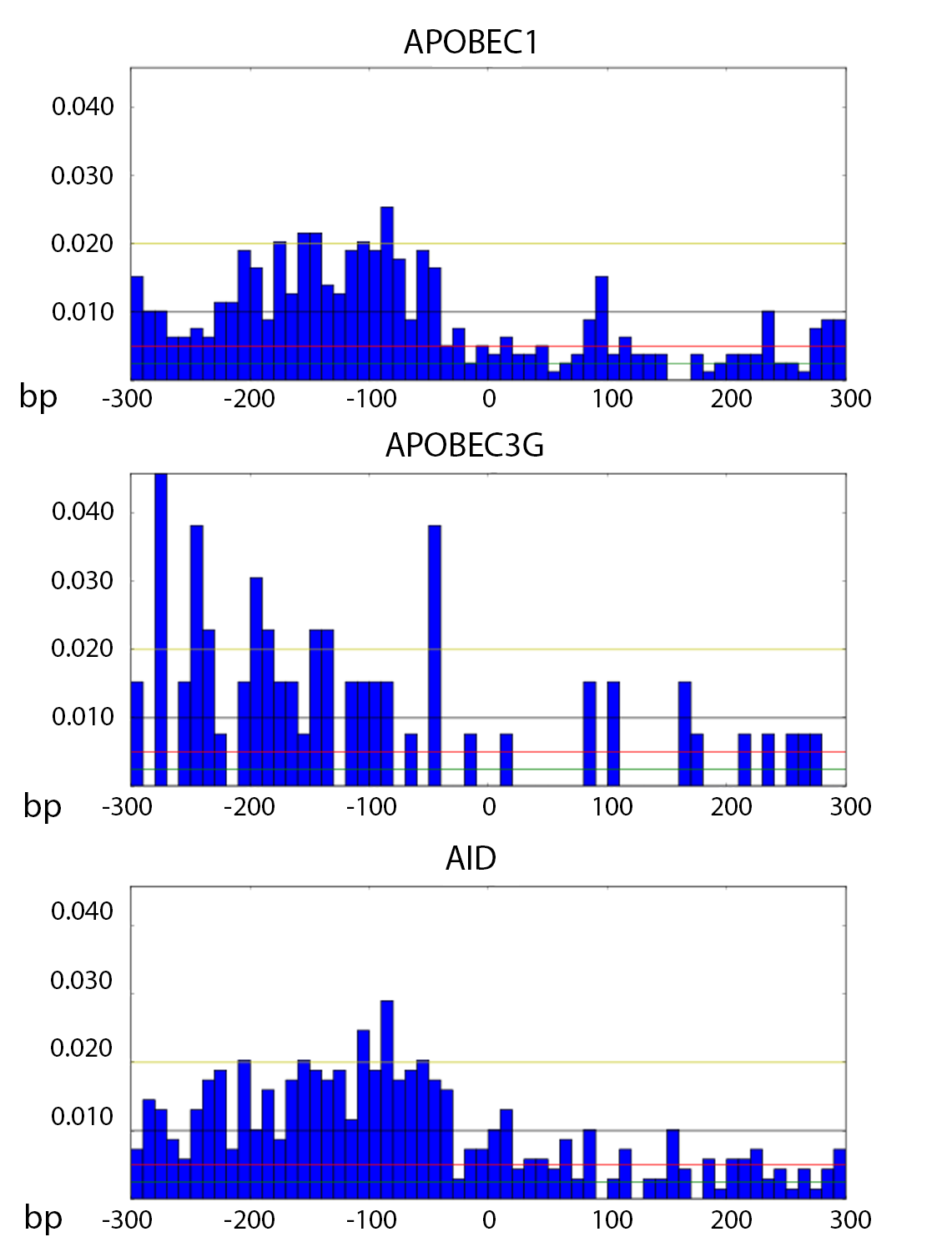

Supplement: S3 Fig — (TIF) [file pgen.1005217.s006.tif]

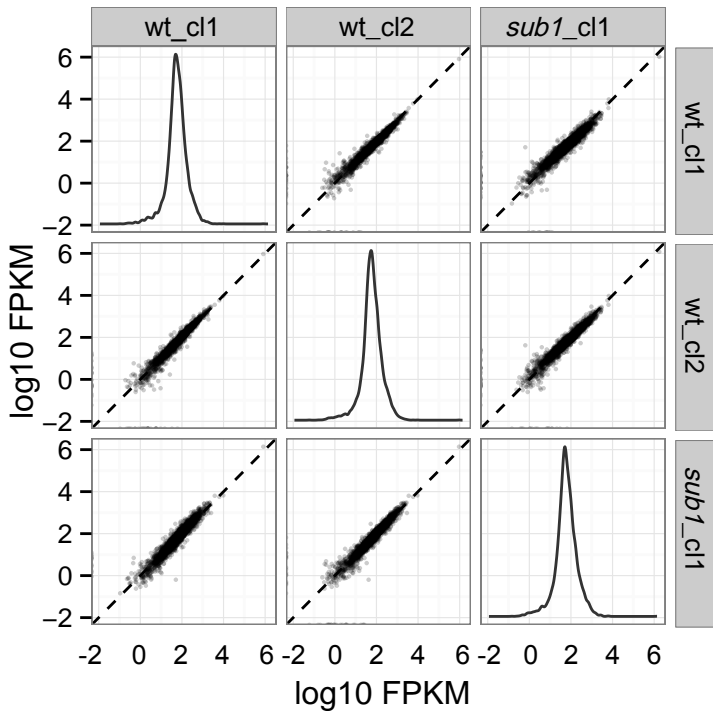

Supplement: S4 Fig — Name explanation: wt_cl1 means wild-type (SUB1) clone 1. (PDF) [file pgen.1005217.s007.pdf]

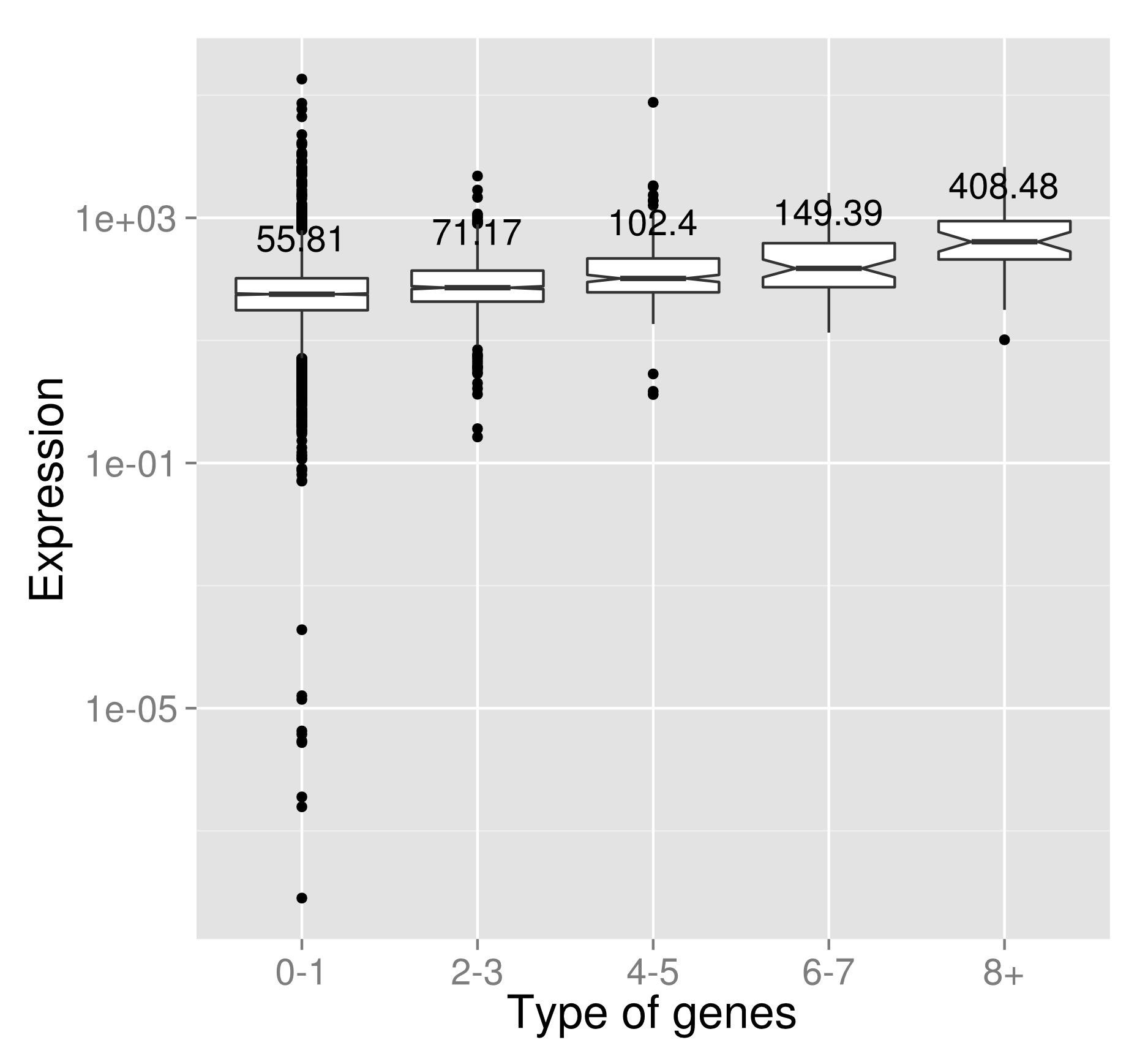

Supplement: S5 Fig — Y-axis, log10(FKPM) values. Other elements of the graph are as described in the legend to Fig 8. (TIF) [file pgen.1005217.s008.tif]

## New reference assembling

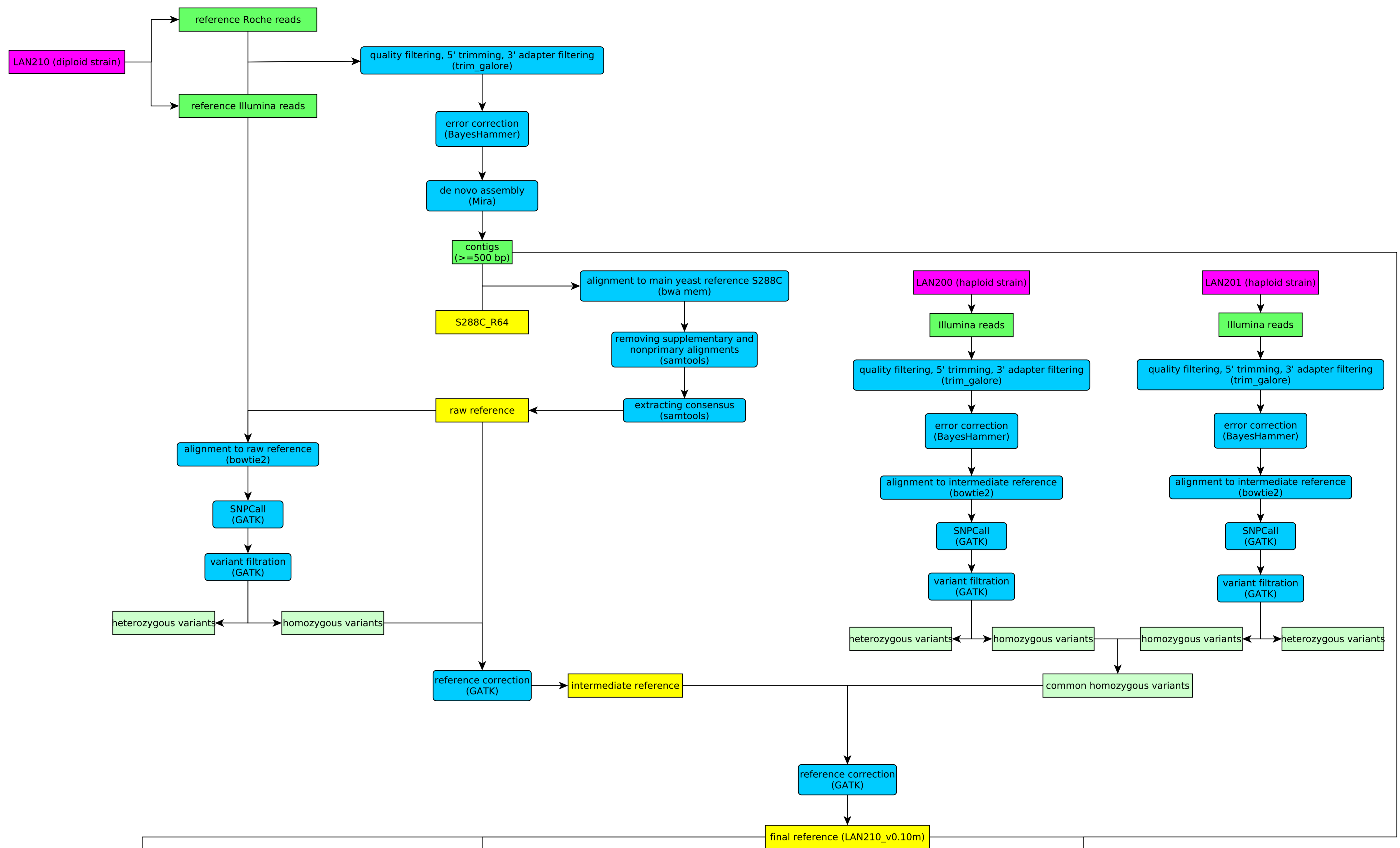

## New reference annotation

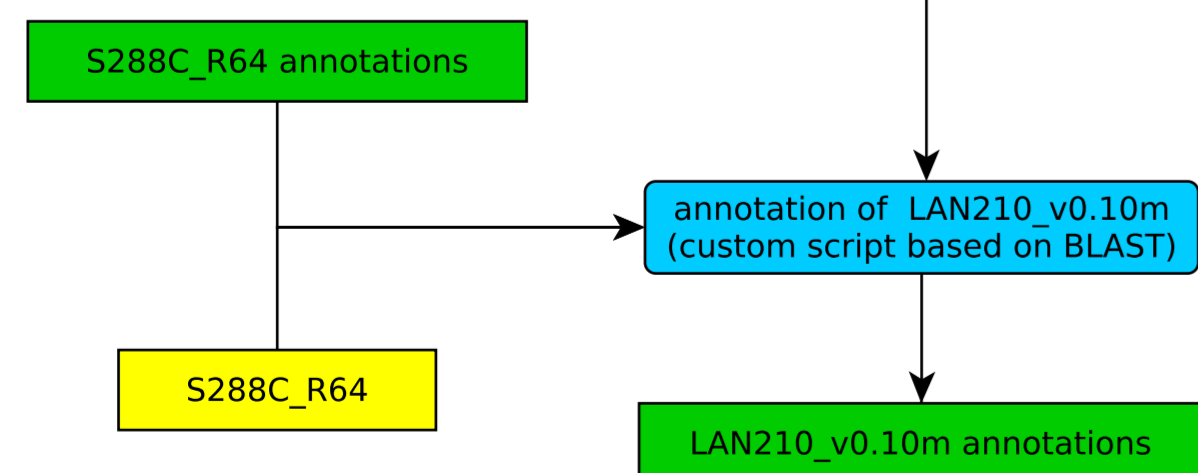

## Reference masking

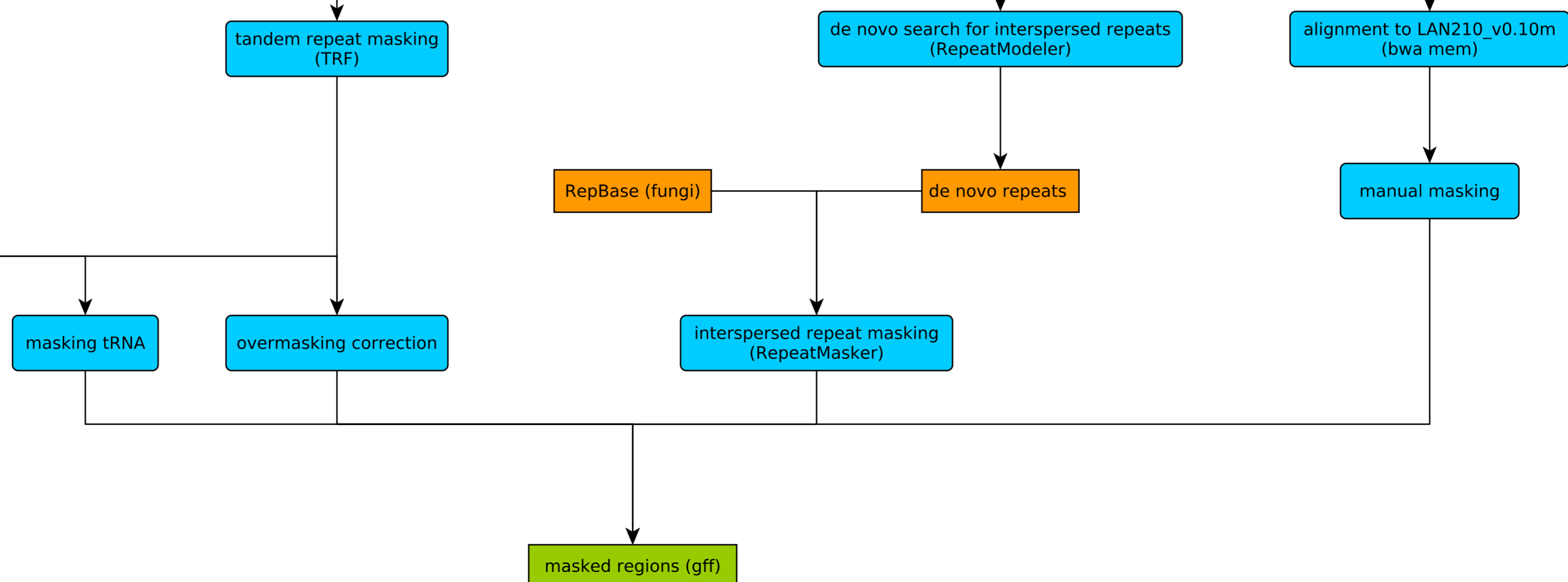

Supplement: S7 Fig — (PDF) [file pgen.1005217.s010.pdf]

SNV call

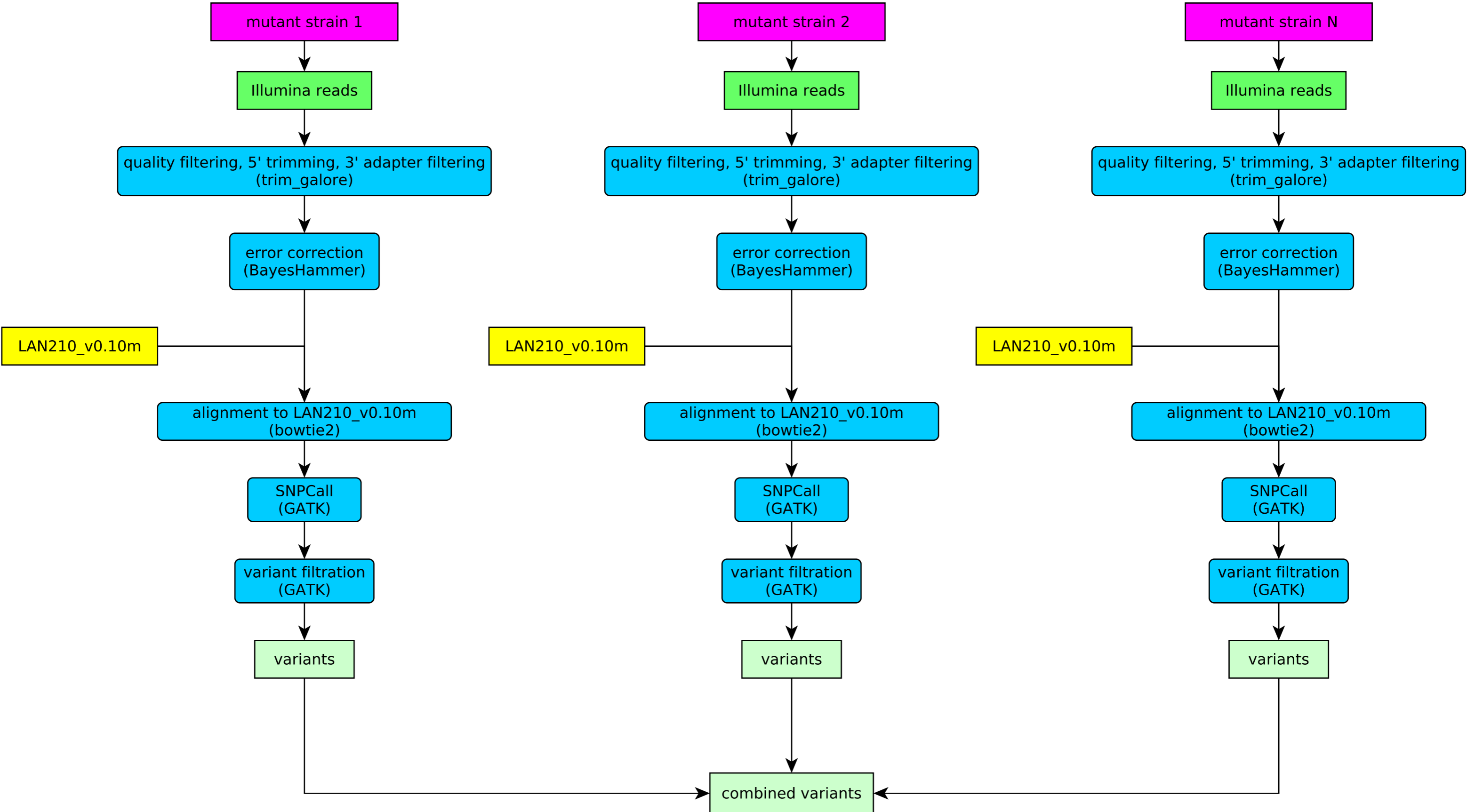

Clustering

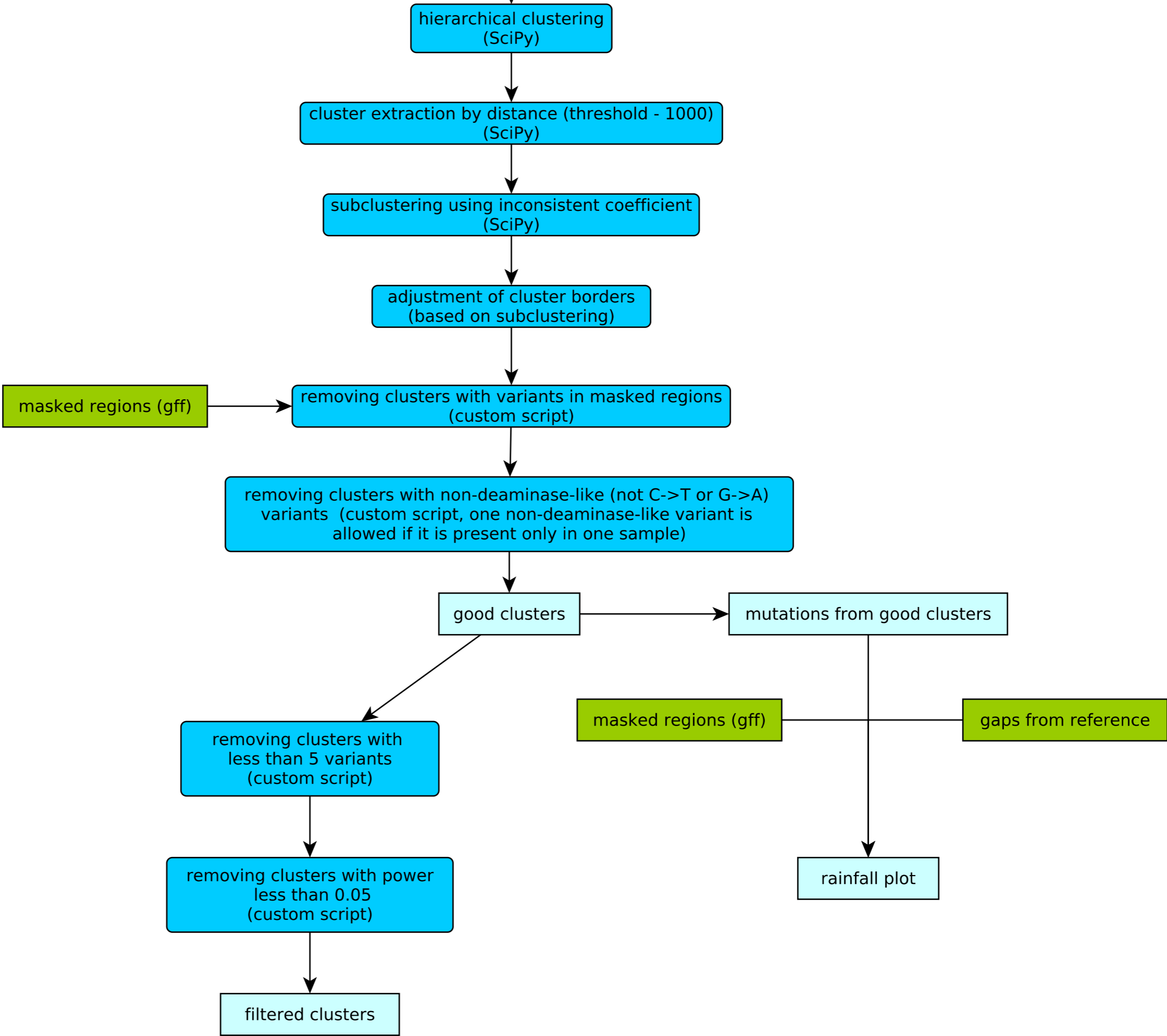

Supplement: S8 Fig — (PDF) [file pgen.1005217.s011.pdf]
