# Supplementary material for: Disruption of Transcriptional Coactivator Sub1 Leads to Genome-Wide Re-distribution of Clustered Mutations Induced by APOBEC in Active Yeast Genes
Source: PLoS Genet. 2015 May 5;11(5):e1005217. doi: 10.1371/journal.pgen.1005217 (PMC4420506; doi:10.1371/journal.pgen.1005217)

6-HAP

Relationship between number of clusters and threshold of distance

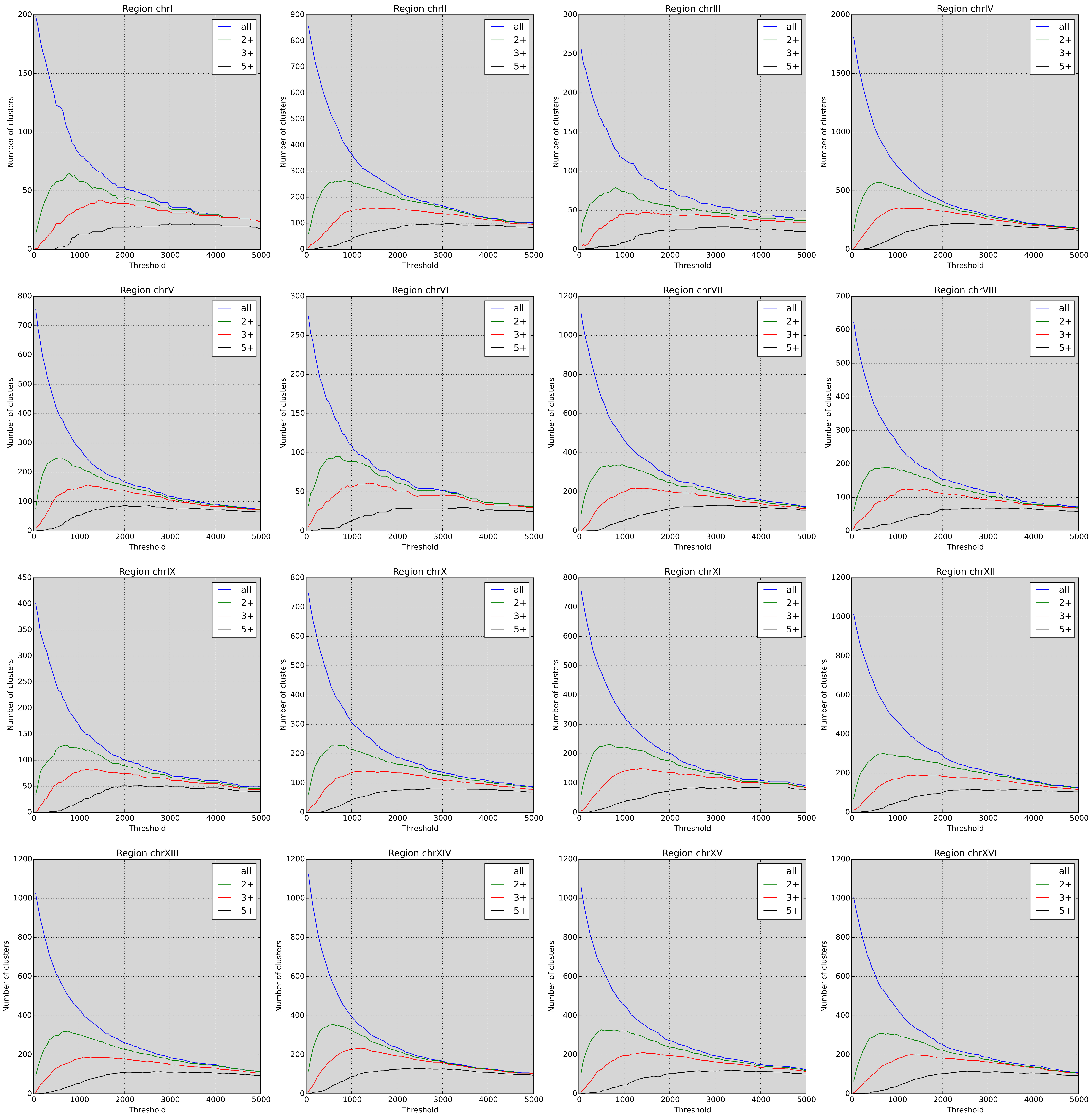

PmCDA1

Relationship between number of clusters and threshold of distance

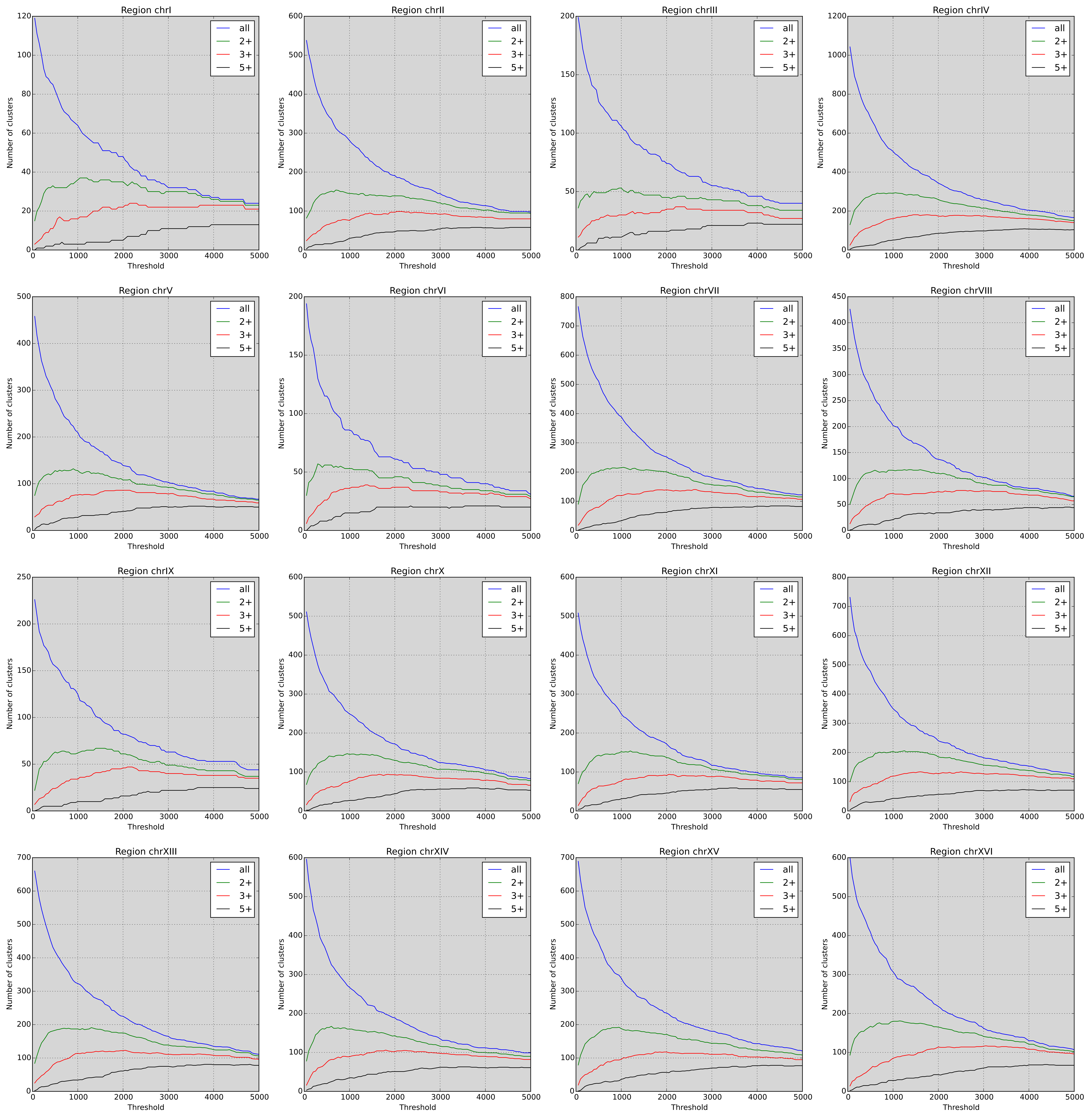

Supplement: S2 Fig — Results for 6-HAP and PmCDA1 data for the clusters with different sizes (number of SNVs) are shown. (PDF) [file pgen.1005217.s005.pdf]
