# Supplementary material for: Disruption of Transcriptional Coactivator Sub1 Leads to Genome-Wide Re-distribution of Clustered Mutations Induced by APOBEC in Active Yeast Genes
Source: PLoS Genet. 2015 May 5;11(5):e1005217. doi: 10.1371/journal.pgen.1005217 (PMC4420506; doi:10.1371/journal.pgen.1005217)
Supplement: S6 Fig — First page. Location of patches with different strains is shown on the scheme at the right of the page. SC-His, synthetic complete media without histidine; SC-Arg+CAN, synthetic complete media without arginine and containing canavanine. Second page. Results on synthetic complete media plates without adenine, tryptophan and lysine (SC-Ade, SC-Trp, and SC-Lys, respectively). The pattern of strain patches is the same as on page 1. (PDF) [file pgen.1005217.s009.pdf]

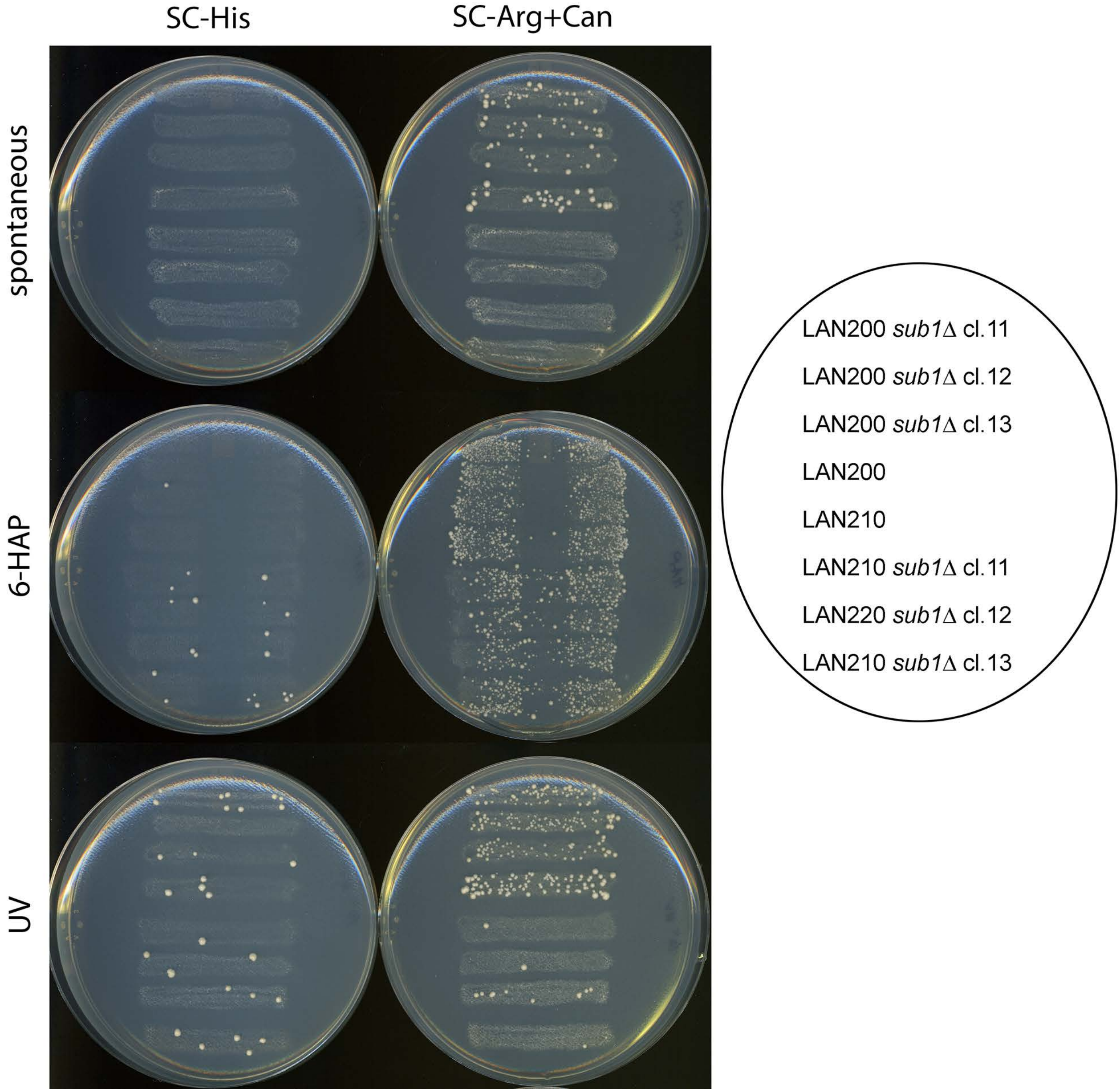
SC-His
SC-Arg+Can
spontaneous
6-HAP
UV
LAN200 *sub1Δ* cl.11
LAN200 *sub1Δ* cl.12
LAN200 *sub1Δ* cl.13
LAN200
LAN210
LAN210 *sub1Δ* cl.11
LAN220 *sub1Δ* cl.12
LAN210 *sub1Δ* cl.13

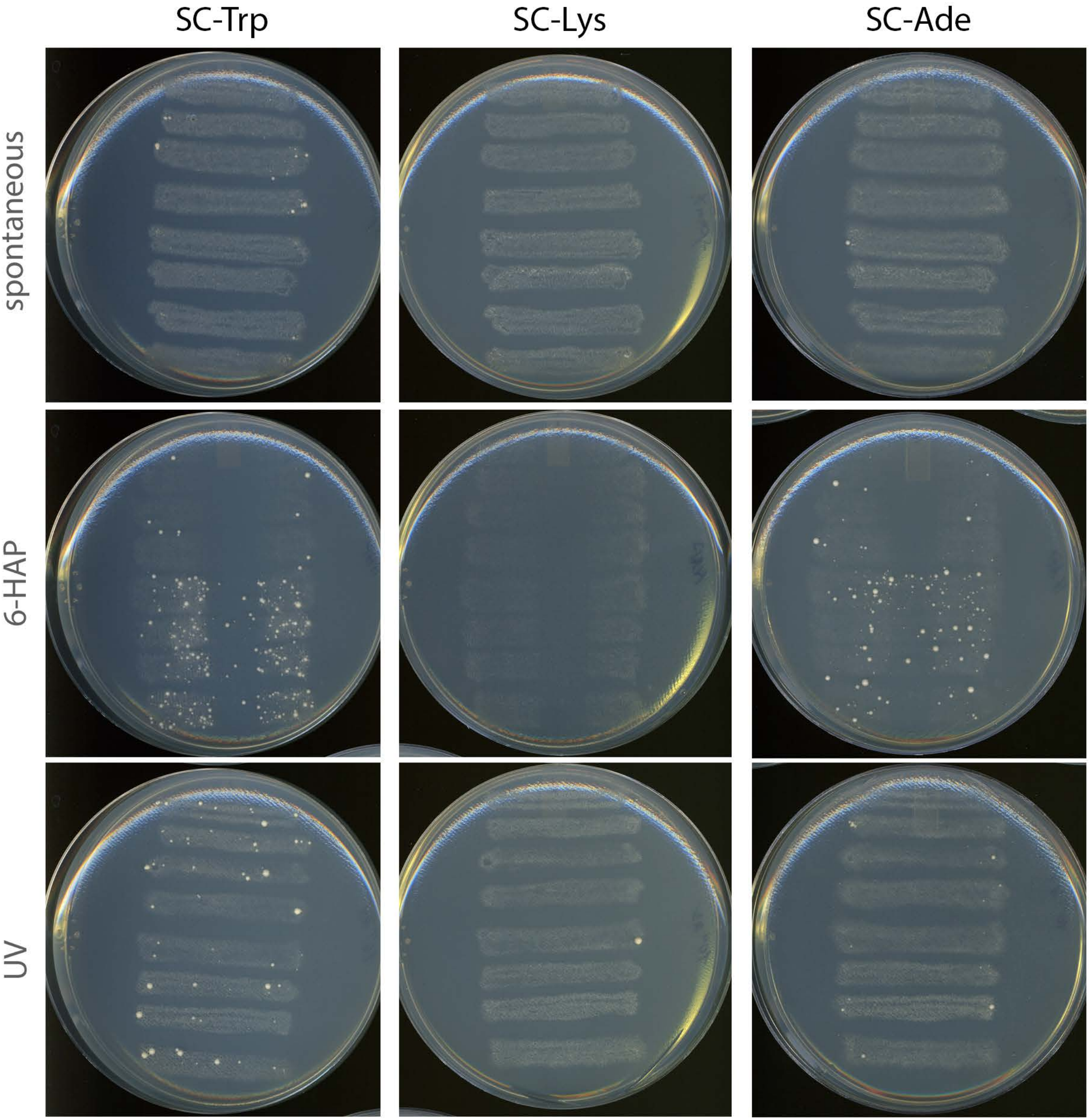
SC-Trp
SC-Lys
SC-Ade
spontaneous
6-HAP
UV
