# Supplementary material for: Disruption of Transcriptional Coactivator Sub1 Leads to Genome-Wide Re-distribution of Clustered Mutations Induced by APOBEC in Active Yeast Genes
Source: PLoS Genet. 2015 May 5;11(5):e1005217. doi: 10.1371/journal.pgen.1005217 (PMC4420506; doi:10.1371/journal.pgen.1005217)
Supplement: S2 Protocol — (ZIP) [file pgen.1005217.s013.zip › CDS_region_vars_2.py]

MAVR/CDS\_region\_vars\_2.py at master · mahajrod/MAVR · GitHub


Skip to content

Sign up
Sign in

This repository

- Explore
- Features
- Enterprise
- Blog

- Star

  0
- Fork

  0

# mahajrod/**MAVR**

- Code
- Issues
- Pull Requests

- Pulse
- Graphs

### HTTPS clone URL

### Subversion checkout URL

You can clone with
HTTPS or Subversion.

Download ZIP

Permalink


*branch:*
master

Switch branches/tags

- Branches
- Tags

master

Nothing to show

Nothing to show

MAVR/examples/desaminases/**CDS\_region\_vars\_2.py**

mahajrod
Dec 26, 2014

A lot of changes

**1**
contributor

## Users who have contributed to this file

- mahajrod

90 lines (81 sloc)

4.016 kb

Raw
Blame
History

|  |  |
| --- | --- |
|  | #!/usr/bin/env python |
|  | \_\_author\_\_ = 'mahajrod' |
|  |  |
|  | import os |
|  | from collections import OrderedDict |
|  | from Parser.VCF import CollectionVCF |
|  | import numpy as np |
|  | import matplotlib.pyplot as plt |
|  |  |
|  | import pprint |
|  | from BCBio.GFF import GFFExaminer |
|  | from BCBio import GFF |
|  |  |
|  | if \_\_name\_\_ == "\_\_main\_\_": |
|  | workdir = "/media/mahajrod/d9e6e5ee-1bf7-4dba-934e-3f898d9611c8/Data/LAN2xx/combined\_vcf/clusters/all/all/" |
|  |  |
|  | sample\_set\_names\_list = ["PmCDA1\_3d", |
|  | "HAP", |
|  | "PmCDA1\_sub1\_3d", |
|  | "PmCDA1\_6d", |
|  | "HAP\_sub1", |
|  | "PmCDA1\_sub1\_6d", |
|  | "A1\_3d", |
|  | "A1\_6d", |
|  | "A3G\_3d", |
|  | "AID\_3d", |
|  | "AID\_6d" |
|  | ] |
|  |  |
|  | annotations = "/home/mahajrod/genetics/desaminases/data/LAN210\_v0.10m/annotations/merged\_annotations\_Nagalakshmi\_tranf\_to\_LAN210\_v0.10m.gff3" |
|  | with open(annotations, "r") as in\_fd: |
|  | record\_dict = dict([(record.id, record) for record in GFF.parse(in\_fd)]) |
|  | start\_dict = OrderedDict({}) |
|  | end\_dict = OrderedDict({}) |
|  | position\_dict = OrderedDict({}) |
|  | length\_dict = OrderedDict({}) |
|  |  |
|  | start\_hist\_dict = OrderedDict({}) |
|  | end\_hist\_dict = OrderedDict({}) |
|  |  |
|  | os.chdir(workdir) |
|  |  |
|  | skip\_genes\_without\_five\_utr = True |
|  | left = 300 |
|  | right = 300 |
|  | bin\_width = 10 |
|  | bins = np.linspace(-left, right, ((left+right)/bin\_width) + 1) |
|  | normed = True |
|  | max\_start = 0 |
|  | max\_end = 0 |
|  | for sample\_set in sample\_set\_names\_list: |
|  | vcf\_file = "%s\_good.vcf" % sample\_set |
|  | #start\_hist\_prefix = "%s\_start\_hist\_r\_%i\_l\_%i" % (sample\_set, right, left) |
|  | #end\_hist\_prefix = "%s\_end\_hist\_r\_%i\_l\_%i" % (sample\_set, right, left) |
|  | #gene\_variants = "%s\_gene\_variants\_r\_%i\_l\_%i.t" % (sample\_set, right, left) |
|  | variants = CollectionVCF(from\_file=True, vcf\_file=vcf\_file) |
|  | start\_dict[sample\_set], end\_dict[sample\_set], position\_dict[sample\_set] = \ |
|  | variants.variants\_start\_end(left, right, record\_dict, skip\_genes\_without\_five\_utr=skip\_genes\_without\_five\_utr) |
|  | length\_dict[sample\_set] = len(variants) |
|  | #print(start\_dict[sample\_set]) |
|  | start\_hist\_dict[sample\_set] = list(np.histogram(start\_dict[sample\_set], bins=bins)) |
|  | end\_hist\_dict[sample\_set] = list(np.histogram(end\_dict[sample\_set], bins=bins)) |
|  | print(start\_hist\_dict[sample\_set][0]) |
|  | if normed: |
|  | start\_hist\_dict[sample\_set][0] = start\_hist\_dict[sample\_set][0].astype(np.float32, copy=False) |
|  | end\_hist\_dict[sample\_set][0] = end\_hist\_dict[sample\_set][0].astype(np.float32, copy=False) |
|  | start\_hist\_dict[sample\_set][0] = start\_hist\_dict[sample\_set][0] / length\_dict[sample\_set] |
|  | end\_hist\_dict[sample\_set][0] = end\_hist\_dict[sample\_set][0] / length\_dict[sample\_set] |
|  | print("Normed") |
|  | print(start\_hist\_dict[sample\_set][0]) |
|  | max\_end = max(max\_end, np.amax(end\_hist\_dict[sample\_set][0])) |
|  | max\_start = max(max\_start, np.amax(start\_hist\_dict[sample\_set][0])) |
|  | plt.figure(1, dpi=300, figsize=(16, 8\*len(sample\_set\_names\_list))) |
|  |  |
|  | index = 1 |
|  | for sample\_set in sample\_set\_names\_list: |
|  | plt.subplot(len(sample\_set\_names\_list), 1, index) |
|  | plt.bar(start\_hist\_dict[sample\_set][1][:-1], start\_hist\_dict[sample\_set][0], width=bin\_width) |
|  | plt.xlim(xmin=-left, xmax=right) |
|  | plt.ylim(ymax=max\_start) |
|  | plt.axhline(0.02, color='y') |
|  | plt.axhline(0.01, color='k') |
|  | plt.axhline(0.005, color='r') |
|  | plt.axhline(0.0025, color='g') |
|  | plt.title(sample\_set) |
|  | index += 1 |
|  | skip = "\_skipped\_non\_five\_utr\_genes" if skip\_genes\_without\_five\_utr else "" |
|  | plt.savefig("all\_DA\_CDS\_start\_all\_r\_%i\_l\_%i\_bin\_width\_%i%s.svg" % (right, left, bin\_width, skip)) |
|  | plt.savefig("all\_DA\_CDS\_start\_all\_r\_%i\_l\_%i\_bin\_width\_%i%s.eps" % (right, left, bin\_width, skip)) |
|  | plt.close() |

Jump to Line

Go

- Status
- API
- Training
- Shop
- Blog
- About


- © 2015 GitHub, Inc.
- Terms
- Privacy
- Security
- Contact

Something went wrong with that request. Please try again.
