# Supplementary material for: Disruption of Transcriptional Coactivator Sub1 Leads to Genome-Wide Re-distribution of Clustered Mutations Induced by APOBEC in Active Yeast Genes
Source: PLoS Genet. 2015 May 5;11(5):e1005217. doi: 10.1371/journal.pgen.1005217 (PMC4420506; doi:10.1371/journal.pgen.1005217)
Supplement: S2 Protocol — (ZIP) [file pgen.1005217.s013.zip › TSS_UTR_CDS_histogram.py]

MAVR/TSS\_UTR\_CDS\_histogram.py at master · mahajrod/MAVR · GitHub


Skip to content

Sign up
Sign in

This repository

- Explore
- Features
- Enterprise
- Blog

- Star

  0
- Fork

  0

# mahajrod/**MAVR**

- Code
- Issues
- Pull Requests

- Pulse
- Graphs

### HTTPS clone URL

### Subversion checkout URL

You can clone with
HTTPS or Subversion.

Download ZIP

Permalink


*branch:*
master

Switch branches/tags

- Branches
- Tags

master

Nothing to show

Nothing to show

MAVR/examples/desaminases/**TSS\_UTR\_CDS\_histogram.py**

Fetching contributors…

Cannot retrieve contributors at this time

191 lines (167 sloc)

8.825 kb

Raw
Blame
History

|  |  |
| --- | --- |
|  | #!/usr/bin/env python |
|  | \_\_author\_\_ = 'mahajrod' |
|  |  |
|  | import os |
|  | from collections import OrderedDict |
|  | from Parser.VCF import CollectionVCF |
|  | import numpy as np |
|  | import matplotlib.pyplot as plt |
|  |  |
|  | import pprint |
|  | from BCBio.GFF import GFFExaminer |
|  | from BCBio import GFF |
|  |  |
|  |  |
|  | def variants\_start\_end(collection, left, right, record\_dict, min\_five\_utr\_len=10, skip\_nonintergenic\_variants=False): |
|  |  |
|  | pre\_UTR\_positions = [] |
|  | UTR\_positions = [] |
|  | CDS\_positions = [] |
|  |  |
|  | for record\_id in record\_dict: |
|  | for feature in record\_dict[record\_id].features: |
|  | if feature.type != "gene": |
|  | continue |
|  |  |
|  | for sub\_feature in feature.sub\_features: |
|  | if sub\_feature.type == "five\_prime\_UTR" and len(sub\_feature) >= min\_five\_utr\_len: |
|  | break |
|  | else: |
|  | continue |
|  | #print(feature.sub\_features) |
|  | for sub\_feature in feature.sub\_features: |
|  | strand = sub\_feature.strand |
|  | if sub\_feature.type == "five\_prime\_UTR": |
|  | five\_UTR\_start = sub\_feature.location.start + 1 if strand == +1 else sub\_feature.location.end |
|  | pre\_UTR\_start = five\_UTR\_start - left if strand == +1 else five\_UTR\_start + 1 |
|  | pre\_UTR\_end = five\_UTR\_start - 1 if strand == +1 else five\_UTR\_start + left |
|  |  |
|  | for variant in collection: |
|  | if record\_id != variant.chrom: |
|  | continue |
|  | if variant.pos - 1 in sub\_feature: |
|  | #print(five\_UTR\_start) |
|  | #print(variant) |
|  | relative\_position = float((variant.pos - five\_UTR\_start) \* strand) \* 100 / len(sub\_feature) |
|  | if relative\_position < 0: |
|  | print(variant) |
|  | print(sub\_feature) |
|  | UTR\_positions.append(relative\_position) |
|  | elif pre\_UTR\_start <= variant.pos <= pre\_UTR\_end: |
|  | if skip\_nonintergenic\_variants and variant.info\_dict["Ftype"] != ["igc"]: |
|  | continue |
|  |  |
|  | relative\_position = (variant.pos - five\_UTR\_start) \* strand |
|  | if relative\_position > 0: |
|  | print(pre\_UTR\_start, pre\_UTR\_end, five\_UTR\_start) |
|  | print(variant) |
|  | print(sub\_feature) |
|  | pre\_UTR\_positions.append(relative\_position) |
|  |  |
|  | continue |
|  | if sub\_feature.type != "CDS": |
|  | continue |
|  | strand = sub\_feature.strand |
|  | CDS\_start = sub\_feature.location.start + 1 if strand == +1 else sub\_feature.location.end |
|  |  |
|  | region\_start\_start = CDS\_start if strand == +1 else CDS\_start - right |
|  | region\_start\_end = CDS\_start + right if strand == +1 else CDS\_start |
|  |  |
|  | for variant in collection: |
|  | if record\_id != variant.chrom: |
|  | continue |
|  | if region\_start\_start <= variant.pos <= region\_start\_end: |
|  | CDS\_positions.append((variant.pos - CDS\_start) \* strand) |
|  |  |
|  | return pre\_UTR\_positions, UTR\_positions, CDS\_positions |
|  |  |
|  | if \_\_name\_\_ == "\_\_main\_\_": |
|  | workdir = "/media/mahajrod/d9e6e5ee-1bf7-4dba-934e-3f898d9611c8/Data/LAN2xx/combined\_vcf/clusters/all/all/" |
|  |  |
|  | sample\_set\_names\_list = ["PmCDA1\_3d", |
|  | "HAP", |
|  | "PmCDA1\_sub1\_3d", |
|  | "PmCDA1\_6d", |
|  | "HAP\_sub1", |
|  | "PmCDA1\_sub1\_6d", |
|  | #"A1\_3d", |
|  | #"A1\_6d", |
|  | #"A3G\_3d", |
|  | #"AID\_3d", |
|  | #"AID\_6d" |
|  | ] |
|  |  |
|  | annotations = "/home/mahajrod/genetics/desaminases/data/LAN210\_v0.10m/annotations/merged\_annotations\_Nagalakshmi\_tranf\_to\_LAN210\_v0.10m.gff3" |
|  | with open(annotations, "r") as in\_fd: |
|  | record\_dict = dict([(record.id, record) for record in GFF.parse(in\_fd)]) |
|  | pre\_UTR\_positions = OrderedDict({}) |
|  | UTR\_positions = OrderedDict({}) |
|  | length\_dict = OrderedDict({}) |
|  | CDS\_positions = OrderedDict({}) |
|  |  |
|  | pre\_UTR\_hist\_dict = OrderedDict({}) |
|  | UTR\_hist\_dict = OrderedDict({}) |
|  | CDS\_hist\_dict = OrderedDict({}) |
|  | os.chdir(workdir) |
|  |  |
|  | left = 300 |
|  | right = 300 |
|  | bin\_width = 5 |
|  | pre\_UTR\_bins = left / bin\_width |
|  | CDS\_bins = np.linspace(0, right, right / bin\_width + 1) |
|  | UTR\_bins = 10 |
|  | normed = True |
|  | max\_start = 0 |
|  | max\_end = 0 |
|  | skip\_nonintergenic\_variants = True |
|  | for sample\_set in sample\_set\_names\_list: |
|  | vcf\_file = "%s\_good.vcf" % sample\_set |
|  | #start\_hist\_prefix = "%s\_start\_hist\_r\_%i\_l\_%i" % (sample\_set, right, left) |
|  | #end\_hist\_prefix = "%s\_end\_hist\_r\_%i\_l\_%i" % (sample\_set, right, left) |
|  | #gene\_variants = "%s\_gene\_variants\_r\_%i\_l\_%i.t" % (sample\_set, right, left) |
|  | variants = CollectionVCF(from\_file=True, vcf\_file=vcf\_file) |
|  | pre\_UTR\_positions[sample\_set], UTR\_positions[sample\_set], CDS\_positions[sample\_set] = \ |
|  | variants\_start\_end(variants, left, right, record\_dict, min\_five\_utr\_len=10, |
|  | skip\_nonintergenic\_variants=skip\_nonintergenic\_variants) |
|  | length\_dict[sample\_set] = len(variants) |
|  | #print(start\_dict[sample\_set]) |
|  | pre\_UTR\_hist\_dict[sample\_set] = list(np.histogram(pre\_UTR\_positions[sample\_set], bins=pre\_UTR\_bins)) |
|  | UTR\_hist\_dict[sample\_set] = list(np.histogram(UTR\_positions[sample\_set], bins=UTR\_bins)) |
|  | CDS\_hist\_dict[sample\_set] = list(np.histogram(CDS\_positions[sample\_set], bins=CDS\_bins)) |
|  | print("UTR") |
|  | print(UTR\_positions[sample\_set]) |
|  | print("blablabla") |
|  | print(pre\_UTR\_hist\_dict[sample\_set][0]) |
|  | print(UTR\_hist\_dict[sample\_set][0]) |
|  | print(CDS\_hist\_dict[sample\_set][0]) |
|  | if normed: |
|  | pre\_UTR\_hist\_dict[sample\_set][0] = pre\_UTR\_hist\_dict[sample\_set][0].astype(np.float32, copy=False) |
|  | UTR\_hist\_dict[sample\_set][0] = UTR\_hist\_dict[sample\_set][0].astype(np.float32, copy=False) |
|  | CDS\_hist\_dict[sample\_set][0] = CDS\_hist\_dict[sample\_set][0].astype(np.float32, copy=False) |
|  |  |
|  | pre\_UTR\_hist\_dict[sample\_set][0] = pre\_UTR\_hist\_dict[sample\_set][0] / length\_dict[sample\_set] |
|  | UTR\_hist\_dict[sample\_set][0] = UTR\_hist\_dict[sample\_set][0] / length\_dict[sample\_set] |
|  | CDS\_hist\_dict[sample\_set][0] = CDS\_hist\_dict[sample\_set][0] / length\_dict[sample\_set] |
|  |  |
|  | print("Normed") |
|  | print(pre\_UTR\_hist\_dict[sample\_set][0]) |
|  | print(UTR\_hist\_dict[sample\_set][0]) |
|  | print(CDS\_hist\_dict[sample\_set][0]) |
|  | max\_start = max(max\_start, np.amax(pre\_UTR\_hist\_dict[sample\_set][0]), |
|  | np.amax(UTR\_hist\_dict[sample\_set][0]), |
|  | np.amax(CDS\_hist\_dict[sample\_set][0]) ) |
|  | plt.figure(1, dpi=300, figsize=(24, 8\*len(sample\_set\_names\_list))) |
|  |  |
|  | index = 0 |
|  | for sample\_set in sample\_set\_names\_list: |
|  | plt.subplot(len(sample\_set\_names\_list), 3, index \* 3 + 1) |
|  | plt.bar(pre\_UTR\_hist\_dict[sample\_set][1][:-1], pre\_UTR\_hist\_dict[sample\_set][0], width=bin\_width) |
|  | plt.xlim(xmin=-left, xmax=0) |
|  | plt.ylim(ymax=max\_start) |
|  | plt.axhline(0.02, color='y') |
|  | plt.axhline(0.01, color='k') |
|  | plt.axhline(0.005, color='r') |
|  | plt.axhline(0.0025, color='g') |
|  | plt.title(sample\_set + " pre 5' UTR") |
|  |  |
|  | plt.subplot(len(sample\_set\_names\_list), 3, index \* 3 + 2) |
|  | plt.bar(UTR\_hist\_dict[sample\_set][1][:-1], UTR\_hist\_dict[sample\_set][0], width=10) |
|  | plt.xlim(xmin=0, xmax=100) |
|  | plt.ylim(ymax=max\_start) |
|  | plt.axhline(0.02, color='y') |
|  | plt.axhline(0.01, color='k') |
|  | plt.axhline(0.005, color='r') |
|  | plt.axhline(0.0025, color='g') |
|  | plt.title(sample\_set + " 5' UTR (% pos)") |
|  |  |
|  | plt.subplot(len(sample\_set\_names\_list), 3, index \* 3 + 3) |
|  | plt.bar(CDS\_hist\_dict[sample\_set][1][:-1], CDS\_hist\_dict[sample\_set][0], width=bin\_width) |
|  | plt.xlim(xmin=0, xmax=right) |
|  | plt.ylim(ymax=max\_start) |
|  | plt.axhline(0.02, color='y') |
|  | plt.axhline(0.01, color='k') |
|  | plt.axhline(0.005, color='r') |
|  | plt.axhline(0.0025, color='g') |
|  | plt.title(sample\_set + " CDS") |
|  | index += 1 |
|  |  |
|  | suffix = "pre\_five\_UTR\_only\_intergenic" if skip\_nonintergenic\_variants else "all" |
|  | plt.savefig("TSS\_UTR\_CDS\_start\_all\_r\_%i\_l\_%i\_bin\_width\_%i\_%s.svg" % (right, left, bin\_width, suffix)) |
|  | plt.savefig("TSS\_UTR\_CDS\_start\_all\_r\_%i\_l\_%i\_bin\_width\_%i\_%s.eps" % (right, left, bin\_width, suffix)) |
|  | plt.close() |

Jump to Line

Go

- Status
- API
- Training
- Shop
- Blog
- About


- © 2015 GitHub, Inc.
- Terms
- Privacy
- Security
- Contact

Something went wrong with that request. Please try again.
